# Contribution to the knowledge of Afrotropical Dryinidae, Embolemidae and Sclerogibbidae (Hymenoptera), with description of new species from Central African Republic and Uganda

**DOI:** 10.3897/zookeys.578.7820

**Published:** 2016-04-07

**Authors:** Massimo Olmi, Simon van Noort, Adalgisa Guglielmino

**Affiliations:** 1 Tropical Entomology Research Center, Viterbo, Via De Gasperi 10, 01100 Italy; 2 Natural History Department, Iziko South African Museum, PO Box 61, Cape Town, 8000, South Africa; 3 Department of Biological Sciences, University of Cape Town, Private Bag, Rondebosch, 7701, South Africa; 4 Department of Agriculture, Forests, Nature and Energy, University of Tuscia, Via San Camillo de Lellis, Viterbo, 01100 Italy

**Keywords:** Chrysidoidea, new records, taxonomy, biogeography, Afrotropical Region

## Abstract

An updated checklist of Dryinidae, Embolemidae and Sclerogibbidae from Central African Republic and Uganda is presented. The following new species of Dryinidae are described: from Central African Republic: *Anteon dzanganum* **sp. n.** (Anteoninae); from Uganda: *Anteon granulatum* **sp. n.**, *Anteon kibalense* **sp. n.**, *Anteon makererense* **sp. n.**, *Anteon mubfs* **sp. n.** (Anteoninae); *Bocchus kibalensis* **sp. n.** (Bocchinae); *Dryinus kibalus* **sp. n.** (Dryininae); *Gonatopus kanyawarus* **sp. n.** (Gonatopodinae). The following species have been recorded for the first time from Central African Republic: Embolemidae: *Ampulicomorpha madecassa* Olmi, 1999a; *Embolemus capensis* Olmi, 1997; Dryinidae: *Aphelopus mediocarinatus* (Benoit, 1951d), *Aphelopus testaceus* Olmi, 1991, *Aphelopus wittei* Benoit, 1951c (Aphelopinae); *Anteon cautum* Olmi, 1994a, *Anteon evertsi* Olmi, 1989, *Anteon gutturnium* (Benoit, 1951b), *Anteon inflatrix* Benoit, 1951b, *Anteon kivuanum* (Benoit, 1951c), *Anteon semajanna* Olmi, Copeland & Guglielmino, 2015, *Anteon zairense* Benoit, 1951d (Anteoninae); *Pseudodryinus townesi* (Olmi, 1984) (Dryininae); *Echthrodelphax tauricus* Ponomarenko, 1970, *Gonatopus camerounensis* Olmi, 2011, *Gonatopus kolyadai* Olmi, 2007b, *Neodryinus antiquus* Benoit, 1954, *Neodryinus tussaci* Olmi, 2004b (Gonatopodinae); Sclerogibbidae: *Probethylus callani* Richards, 1939b; *Sclerogibba algerica* Benoit, 1963, *Sclerogibba rapax* Olmi, 2005a. The following species have been recorded for the first time from Uganda: Embolemidae: *Ampulicomorpha magna* Olmi, 1996; Dryinidae: *Anteon cautum* Olmi, 1994a, *Anteon fisheri* Olmi, 2003, *Anteon hoyoi* Olmi, 1984, *Anteon kivuanum* (Benoit, 1951c), *Anteon townesi* Olmi, 1984, *Anteon zairense* Benoit, 1951d (Anteoninae); *Bocchus bini* Olmi, 1984 (Bocchinae); *Dryinus saussurei* (Ceballos, 1936) (Dryininae); *Echthrodelphax migratorius* Benoit, 1954, *Neodryinus tussaci* Olmi, 2004b (Gonatopodinae). The following further species has been recorded for the first time from Mali: *Sclerogibba algerica* Benoit, 1963 (Sclerogibbidae); from Ivory Coast: *Adryinus oweni* Olmi, 1984 (Gonatopodinae); from Cameroon and South Africa: *Gonatopus operosus* Olmi, 1993 (Gonatopodinae); from Democratic Republic of the Congo and Zambia: *Neodryinus antiquus* Benoit, 1954 (Gonatopodinae); from South Africa: *Anteon striatum* Olmi, 2005b (Anteoninae). Including the above new records, 23 species of Dryinidae (previously six), two species of Embolemidae (previously none) and three species of Sclerogibbidae (previously two) are now known from Central African Republic. For Uganda, 39 species of Dryinidae (previously 23), one species of Embolemidae (previously none) and four species of Sclerogibbidae (previously four) are now known. Additional new faunistic records are provided for Cameroon, Democratic Republic of the Congo, Ivory Coast, Mali, South Africa and Zambia.

## Introduction

Dryinidae and Embolemidae (Hymenoptera: Chrysidoidea) are parasitoids of Auchenorrhyncha (Hemiptera) (Guglielmino et al. 2013; Olmi 1996; Olmi et al. 2014). Sclerogibbidae (Hymenoptera: Chrysidoidea) are parasitoids of Embiidina (Olmi 2005a).

Afrotropical species of the above three families are poorly known. In recent years research investigation has been intensified in Burundi, Kenya, Madagascar, Mozambique and South Africa (mainly thanks to the efforts of Robert S. Copeland in Burundi and Kenya, Brian L. Fisher in Madagascar, Massimo Olmi in Mozambique and Simon van Noort in South Africa). The above inventory surveys resulted in the descriptions of many new species, and contributed to the publication of updated checklists (Azevedo et al. 2010 and Olmi 2007a, 2010 on Madagascar; Olmi and Copeland 2011 and Olmi et al. 2015 on Burundi and Kenya; Olmi et al. 2012 on Mozambique; Olmi 2006, 2007b, 2009 on South Africa). However, in spite of the above efforts, the fauna of dryinids, embolemids and sclerogibbids of many countries remains almost unknown.

In addition one of the authors (Simon van Noort) extended his research to two of the lesser known Afrotropical countries, Central African Republic and Uganda, where the number of recorded species was particularly low (in the Central Africa Republic, 6 species of Dryinidae, two species of Sclerogibbidae, no species of Embolemidae; in Uganda, 23 species of Dryinidae, one species of Embolemidae and four species of Sclerogibbidae). The subsequent study of the collected material has resulted in the discovery of eight new species described herein and, along with new distributional data for previously described species of these three families, has provided the opportunity to update the checklist of Dryinids, Embolemids and Sclerogibbids known from Central African Republic and Uganda.

## Material and methods

Species descriptions follow the terminology used by Olmi (1984, 1994c, 1999b), Xu et al. (2013), Olmi and Virla (2014) and Olmi and Xu (2015). The measurements reported are relative, except for the total length (head to abdominal tip, without the antennae), which is expressed in millimetres. In the figures of male genitalia the right half is not included. The following abbreviations are used in the descriptions: POL distance between the inner edges of the two lateral ocelli; OL distance between the inner edges of a lateral ocellus and the median ocellus; OOL distance from the outer edge of a lateral ocellus to the compound eye; OPL distance from the posterior edge of a lateral ocellus to the occipital carina; TL distance from the posterior edge of the eye to the occipital carina.

## Surveyed areas

The Dzanga-Sangha protected area was surveyed in Central African Republic. This area lies north of the equator and is located in the southwest triangle of the country (Sangha-Mbaéré Prefecture) bordered by Cameroon and the Republic of Congo. The Dzanga-Sangha protected area includes the Dzanga-Ndoki National Park (1220 km^2^), and the Dzanga-Sangha Dense Forest Special Reserve (3359 km^2^). The latter is a multiple use zone where logging, traditional hunting, safari hunting and extraction of plants are still allowed under controlled conditions. Annual rainfall is about 1500 mm, with average temperatures ranging between 25°and 29°Celsius. There are two peaks to the rainy season with highest precipitation occurring during the “long rains” from September to November and a second peak during the “short rains” in May and June (Carroll 1997). The eco-region is a part of the Guineo-Congolian lowland rain forest within the Guineo-Congolian regional centre of endemism (White 1983), characterized by the following species: *Entandrophragma congoense* (Meliaceae); *Pentaclethera eetveldeana* (Mimoseae); *Pericopsis elata* (Fabaceae); and *Gilbertiodendron dewevrei* (Fabaceae). The canopy can reach a height of 60 m. The understory is composed of shrubs, lianas and herbs. Harris (2002) recorded 1090 species of vascular plants in the reserve.

Three separate sites within the forest were sampled (named Camps 1-3). Camp 1 was situated at a marsh clearing, Mabéa Bai, in lowland rainforest 21.4km 53°NE Bayanga, 3°02.01'N, 16°24.57'E, 510m, in the Dzanga-Ndoki National. The vegetation in the Bai marsh clearing is dominated by herbaceous plants including abundant sedges (Cyperaceae) and grasses (Graminae). Characteristic trees of the forest margin include *Lophira alata* and *Berlinia grandiflora*. Camp 2 was situated in lowland rainforest on the banks of the Sangha River in the Dzanga-Sangha Dense Forest Special Reserve, 12.7km 326°NW Bayanga, 3°00.27'N, 16°11.55'E, 420m. The river is about 500 meters wide at this point and ranges from a depth of 20 cm at the end of the dry season (around March) to as much as 5 meters during the height of the rainy season in September and October when the forests adjacent to the banks are flooded. This seasonally flooded forest has a complex architecture with 15–25 m trees forming a canopy with occasional emergent trees to 40 m with gaps less common; the understorey consists of small trees (5–10 m) with herbs and lianas common, whereas shrubs are almost absent (Harris 2002). Camp 3 was situated in lowland rainforest about 1 km from the banks of the Sangha River in the Dzanga-Ndoki National Park, 38.6km 173°S Lidjombo, 2°21.60'N, 16°09.20'E, 350m. Sampling was conducted in the seasonally inundated riparian forest (see above for species composition) and in mixed species terra firma forest above the flood plain. Details of the sampled habitats (including photographs of the sampling sites) and sampling effort conducted during the WWF expedition to Central African Republic are presented in Azevedo et al. 2015.

In Uganda surveys were undertaken within the vicinity of the Makerere University Biological Field Station (MUBFS) (0°33.798'N, 30°21.365'E, 1500 m) in the Kanyawara area of Kibale National Park where 12 main survey sites were sampled. The park is located in western Uganda, approximately 24 km from the eastern edge of the Ruwenzori Mountains and encompasses an area of 766 km^2^ and an altitude varying between 1590 m in the north and 1100 m in the south (Chapman et al. 1997). Kibale forest is transitional between lowland rain forest and montane rain forest and is classified as a mid-altitude, moist, evergreen forest with a canopy height typically between 20 and 30 m, but with some trees exceeding 55 m (Chapman et al. 1997, Skorupa 1988, Struhsaker 1997, Wrangham et al. 1994). The park is dominated by moist evergreen forest (57%), with secondary regenerating forest (19%), woodland (4%), grassland (15%), lakes and wetlands (2%), and exotic tree plantations (1%) comprising the remainder (Chapman et al. 1997). Rainfall is bimodal with highest precipitation concentrated during March to May and September to November. Mean annual rainfall totals 1734 mm; mean maximum temperature is 23.7°C; mean minimum temperature is 15.5°C (Rhode et al. 2006). Chapman et al. (1997), Skorupa (1988), and Struhsaker (1997) provide further details of the Kanyawara area.

### Sampling methods

The Malaise traps were constructed to the specifications of the Townes design (Townes 1972), and made with a fine-meshed netting (grid size of 0.2 mm), with black walls and a white roof. Yellow pan traps comprised plastic bowls of 165mm diameter x 40 mm depth, which were placed on the forest floor along a linear transect. Sweeping: The sweep net used for sampling was based on the design of Noyes (1982), with an opening area of c. 1300 cm^2^, and a collecting bag constructed from fine-meshed netting with a grid size of 0.2 mm. The yellow pan trap collection method is indicated by the following abbreviation: YPT.

All types of the Afrotropical species of Dryinidae, Embolemidae and Sclerogibbidae have been examined. The specimens studied for this paper are deposited in the following collections:

AEIC
American Entomological Institute, Gainesville, Florida, USA 


AMNH
American Museum of Natural History, New York, NY, USA 


BMNH
 The Natural History Museum, London, United Kingdom 


BPBM
 Bernice P. Bishop Museum, Honolulu, Hawaii, USA 


CASC
California Academy of Sciences, San Francisco, USA 


CNCI
 Canadian National Collection of Insects, Ottawa, Canada 


IRSN
 Institut Royal de Sciences Naturelles de Belgique, Bruxelles, Belgium 


MSNG
Museo Civico di Storia Naturale “Giacomo Doria” di Genova, Italy 


MNHN
Muséum National d’Histoire Naturelle, Paris, France 


MOLC
 Massimo Olmi collection, c/o Department of Plant Protection, University of Tuscia, Viterbo, Italy 


MRAC
Musée Royal de l’Afrique Centrale, Tervuren, Belgium 


NMSA
Kwazulu-Natal Museum, Pietermaritzburg, KwaZulu-Natal, South Africa 


OLML
Oberösterreichisches Landesmuseum, Linz, Austria 


RMNH
 Rijksmuseum van Natuurlijke Historie, Leiden, The Netherlands 


SAMC
South African Museum, Cape Town, South Africa 


USNM
 National Museum of Natural History, Washington, DC, USA 


UKIC
Department of Entomology, University of Kentucky, Lexington, Kentucky, USA 


The descriptions of most of the new species are based on the study of a single specimen. Specimens of these families are rarely collected and are poorly represented in world collections. The authors are aware that descriptions of new taxa should normally be based on more individuals. However, on the basis of the experience and knowledge of the authors, the new species are sufficiently characterized to justify their descriptions.

## Checklist of Dryinidae, Embolemidae and Sclerogibbidae from Central African Republic and Uganda

An asterisk (*) indicates that specimens are known only from Central African Republic or Uganda, double asterisk (**) indicates the species is newly recorded from Central African Republic or Uganda.

### Family Dryinidae

#### Subfamily Aphelopinae Perkins, 1912

##### Genus *Aphelopus* Dalman

*Aphelopus* Dalman, 1823.

###### Aphelopus himyarita

Taxon classificationAnimaliaHymenopteraDryinidae

Olmi & van Harten

Aphelopus himyarita Olmi & van Harten, 2006: 312.

####### Material examined.

***Published records***. Olmi et al. (2015): **CENTRAL AFRICAN REPUBLIC: *SANGHA-MBAÉRÉ PREFECTURE***: Dzanga-Ndoki National Park, Mabéa Bai,21.4 Km 53°NE Bayanga, 03°02'01"N 16°24'57"E, 510 m, 5–6.V.2001, Malaise trap, lowland rainforest, marsh clearing, S. van Noort leg., 2♂♂ (SAMC); same locality label, 3–4.V.2001, 2♂♂ (SAMC); same locality label, 1–2.v.2001, 1♂ (SAMC); same locality label, 7–8.V.2001, 9♂♂, 3♀♀ (SAMC); Dzanga-Ndoki National Park, 38.6 km 173°S Lidjombo, 2°21.60'N, 16°03.20'E, 350 m, 22.V.2001, sweep, lowland rainforest, S. van Noort leg., 1♀, 2♂♂ (SAMC). **UGANDA: *WESTERN REGION***: Kabarole District, Kibale National Park, Kanyawara, Makerere University Biological Field Station, 0°33.871'S 30°21.355'E, 1495 m, 12–26.VIII.2008, Malaise trap, secondary mid-altitude rainforest, S. van Noort leg., 2♂♂, 1♀ (SAMC); same locality label, 0°33.408'S 30°22.603'E, 1587 m, Malaise trap, degraded mid-altitude rainforest, S. van Noort leg., 3♀♀, 1♂ (SAMC); same locality label, 2–12.VIII.2008, 0°33.784'S 30°22.617'E, 1500 m, Malaise trap, primary mid-altitude rainforest, S. van Noort leg., 2♀♀, 2♂♂ (SAMC); same locality label, 12–26.VIII.2008, 0°33.823'S 30°21.490'E, 1505 m, Malaise trap, primary mid-altitude rainforest, S. van Noort leg., 1♂ (SAMC).

####### Hosts.

Unknown.

####### Distribution.

Afrotropical, recorded from Cameroon, Central African Republic, Ivory Coast, Kenya, Madagascar, Nigeria, South Africa, Uganda and Yemen (Olmi and Copeland 2011; Olmi et al. 2015; Olmi and van Harten 2006).

###### Aphelopus mediocarinatus

Taxon classificationAnimaliaHymenopteraDryinidae

(Benoit)

Antaphelopus mediocarinatus Benoit, 1951d.
Aphelopus mediocarinatus (Benoit): Olmi 1984: 60.

####### Material examined.

***Published records*.** Olmi (1984): **UGANDA: CENTRAL REGION**: Wakiso District, Entebbe (CNCI). ***New records.* CENTRAL AFRICAN REPUBLIC: *SANGHA-MBAÉRÉ PREFECTURE***: Dzanga-Ndoki National Park, Mabéa Bai,21.4 km 53°NE Bayanga, 03°02'01"N 16°24'57"E, 510 m, 3–4.V.2001, Malaise trap, lowland rainforest, marsh clearing, S. van Noort leg., 1♀ (SAMC); Dzanga-Ndoki National Park, 38.6 km 173°S Lidjombo, 2°21.60'N, 16°03.20'E, 350 m, 22.V.2001, sweep, lowland rainforest, S. van Noort leg., 1♂, 4♀♀ (SAMC); Reserve Speciale de Forêt Dense de Dzanga-Sangha, 12.7 km 326°NW Bayanga, 3°00.27'N, 16°11.55'E, 420 m, 13.V.2001, sweep, lowland rainforest, S. van Noort leg., 1♀, 4♂♂ (SAMC).

####### Hosts.

Cicadellidae Typhlocybinae (Guglielmino et al. 2013): in Mozambique (Niassa Province): *Molopopterus alfa* Dworakowska and *Empoascanara ethiopica* Dworakowska; in Nigeria: *Empoasca* sp.

####### Distribution.

Afrotropical, recorded from almost all sub-saharian countries, from Senegal to Somalia, in addition to Madagascar and Yemen (Olmi and Copeland 2011; Olmi et al. 2015; Olmi and van Harten 2006). Newly recorded from Central African Republic here.

###### Aphelopus testaceus

Taxon classificationAnimaliaHymenopteraDryinidae

Olmi**

Aphelopus testaceus Olmi, 1991: 112.

####### Material examined.

***New records.* CENTRAL AFRICAN REPUBLIC: *SANGHA-MBAÉRÉ PREFECTURE***: Dzanga-Ndoki National Park, Mabéa Bai, 21.4 km 53°NE Bayanga, 3°02.01'N, 16°24.57'E, 510 m, 7–8.V.2001, Malaise trap, lowland rainforest, marsh clearing, S. van Noort leg., 1♂ (SAMC); Dzanga-Ndoki National Park, 38.6 km 173°S Lidjombo, 2°21.60'N, 16°03.20'E, 350 m, 22.V.2001, sweep, lowland rainforest, S. van Noort leg., 1♂ (SAMC).

####### Hosts.

Unknown.

####### Distribution.

Democratic Republic of the Congo, South Africa, Tanzania and Yemen (Olmi and van Harten 2000, 2006). Newly recorded from Central African Republic here.

###### Aphelopus wittei

Taxon classificationAnimaliaHymenopteraDryinidae

Benoit**

Aphelopus wittei Benoit, 1951c: 16.

####### Material examined.

***Published records*.** Olmi (1990): **UGANDA: *WESTERN REGION***: Kasese District, Ruwenzori Range, Misigo, 8550 ft., 2–3.VIII.1952, D.S. Fletcher leg., 3♂♂ (2 in BMNH, 1 in BPBM). ***New records.* CENTRAL AFRICAN REPUBLIC: *Nana-Mambéré Prefecture***: 60 km W Bouar, 05°45'N, 15°13'E, 660 m, 23.III.2010, J. Halada leg., 1♂ (OLML); ***SANGHA-MBAÉRÉ PREFECTURE***: Dzanga-Ndoki National Park, Mabéa Bai, 21.4 km 53°NE Bayanga, 3°02.01'N, 16°24.57'E, 510 m, 7–8.V.2001, Malaise trap, lowland rainforest, marsh clearing, S. van Noort leg., 1♀, 4♂♂ (SAMC); Dzanga-Ndoki National Park, 38.6 km 173°S Lidjombo, 2°21.60'N, 16°03.20'E, 350 m, 22.V.2001, sweep, lowland rainforest, S. van Noort leg., 1♀, 12♂♂ (SAMC); Dzanga-Ndoki National Park, Mabéa Bai, 21.4 km 53°NE Bayanga, 3°02.01'N, 16°24.57'E, 510 m, 6.V.2001, sweep, lowland rainforest, marsh clearing, CAR01-S48, S. van Noort leg., 1♂ (SAMC); Reserve Speciale de Forêt Dense de Dzanga-Sangha, 12.7 km 326°NW Bayanga, 3°00.27'N, 16°11.55'E, 420 m, 13.V.2001, sweep, lowland rainforest, S. van Noort leg., 1♀, 8♂♂ (SAMC). **UGANDA: Western Region**: Kabarole District, Kibale Forest, Kanywara (RMNH); Kabarole District, Kibale National Park, Kanyawara, Makerere University Biological Field Station, 0°34.405'N, 30°21.646'E, 1484 m, 12–26.VIII.2008, Malaise trap, primary mid-altitude rainforest, near stream, S. van Noort leg., 1♂ (SAMC); same locality label, 00°33.891'N, 30°21.468'E, 1506 m, 12–26.VIII.2008, 1♀, 5♂♂ (SAMC); same locality label, 00°35.442'N, 30°21.741'E, 1465 m, 10.VIII.2008, sweep, primary mid-altitude rainforest, near stream, S. van Noort leg., 7♂♂ (SAMC); same locality label, 0°33.871'S 30°21.355'E, 1495 m, 12–26.VIII.2008, Malaise trap, secondary mid-altitude rainforest, S. van Noort leg., 2♂♂, 1♀ (SAMC); same locality label, 0°33.408'S 30°22.603'E, 1587 m, Malaise trap, degraded mid-altitude rainforest, S. van Noort leg., 3♂♂ (SAMC); same locality label, 2–12.VIII.2008, 0°33.784'S 30°22.617'E, 1500 m, Malaise trap, primary mid-altitude rainforest, S. van Noort leg., 1♀, 11♂♂ (SAMC); same locality label, 12–26.VIII.2008, 0°33.823'S 30°21.490'E, 1505 m, Malaise trap, primary mid-altitude rainforest, S. van Noort leg., 2♀♀, 2♂♂ (SAMC); same locality label, 2–12.VIII.2008, 0°33.836'S 30°21.700'E, 1523 m, Malaise trap, primary mid-altitude rainforest, S. van Noort leg., 2♀♀, 3♂♂ (SAMC).

####### Hosts.

Cicadellidae Typhlocybinae (Guglielmino et al. 2013): in North Sudan: *Jacobiasca lybica* (Bergevin & Zanon); in Somalia: *Jacobiella facialis* (Jacobi).

####### Distribution.

Afrotropical, recorded from almost all sub-saharian countries, from Senegal to Somalia, in addition to Madagascar and Yemen (Olmi and Copeland 2011; Olmi et al. 2015; Olmi and van Harten 2006). Newly recorded from Central African Republic here.

#### Subfamily Conganteoninae Olmi, 1984

##### Genus *Conganteon* Benoit

*Conganteon* Benoit, 1951c: 11.

###### Conganteon vulcanicum

Taxon classificationAnimaliaHymenopteraDryinidae

Benoit

Conganteon vulcanicus Benoit, 1951c: 12.

####### Material examined.

***Published record*.** Olmi et al. (2015): **UGANDA: *Western Region***: Kibale National Park, Kanyawara, Makerere University Biological Field Station, 0°33.891'N, 30°21.468'E, 1506 m, 4–26.VIII.2008, YPT, primary mid-altitude rainforest, S. van Noort leg., 1♀ (SAMC).

####### Hosts.

Unknown.

####### Distribution.

Democratic Republic of the Congo, Kenya, Madagascar, Rwanda, South Africa, Uganda (Olmi 1984, 1994a; Olmi et al. 2015).

#### Subfamily Anteoninae Perkins, 1912

##### Genus *Anteon* Jurine

*Anteon* Jurine, 1807: 302.

###### Anteon cautum

Taxon classificationAnimaliaHymenopteraDryinidae

Olmi**

Anteon cautum Olmi, 1994a.

####### Material examined.

***New records*. CENTRAL AFRICAN REPUBLIC: *SANGHA-MBAÉRÉ PREFECTURE***: Reserve Speciale de Forêt dense de Dzanga-Sangha, 12.7 km 326°NW Bayanga, 3°00.27'N, 16°11.55'E, 420 m, 17.V.2001, sweep, lowland rainforest, S. van Noort leg., 4♂♂ (SAMC). **UGANDA: *WESTERN REGION***: Kibale National Park, Kanyawara Makerere University Biological Field Station, 0°33.859'S 30°21.630'E, 1505 m, 5–12.VIII.2005, Malaise trap, primary mid-altitude rainforest, S. van Noort leg., 1♂ (SAMC).

####### Hosts.

Unknown.

####### Distribution.

Kenya, Madagascar and South Africa (Olmi 1994a, 2009; Olmi et al. 2015). Newly recorded from Central African Republic and Uganda here.

###### Anteon dzanganum

Taxon classificationAnimaliaHymenopteraDryinidae

sp. n.*

http://zoobank.org/C58A5227-D69A-4416-B21E-3EA1412F26F5

Figs 1A , 4

####### Type material.

HOLOTYPE: ♀, **CENTRAL AFRICAN REPUBLIC: *SANGHA-MBAÉRÉ PREFECTURE***: Réserve Spéciale de Forêt Dense de Dzanga-Sangha, 12.7 km 326°NW Bayanga, 03°00.27'N, 16°11.55'E, 420 m, 11–17.V.2001, YPT, lowland rainforest, CAR01-Y26, S. van Noort leg. (SAMC).

####### Diagnosis.

**Figure 1.**
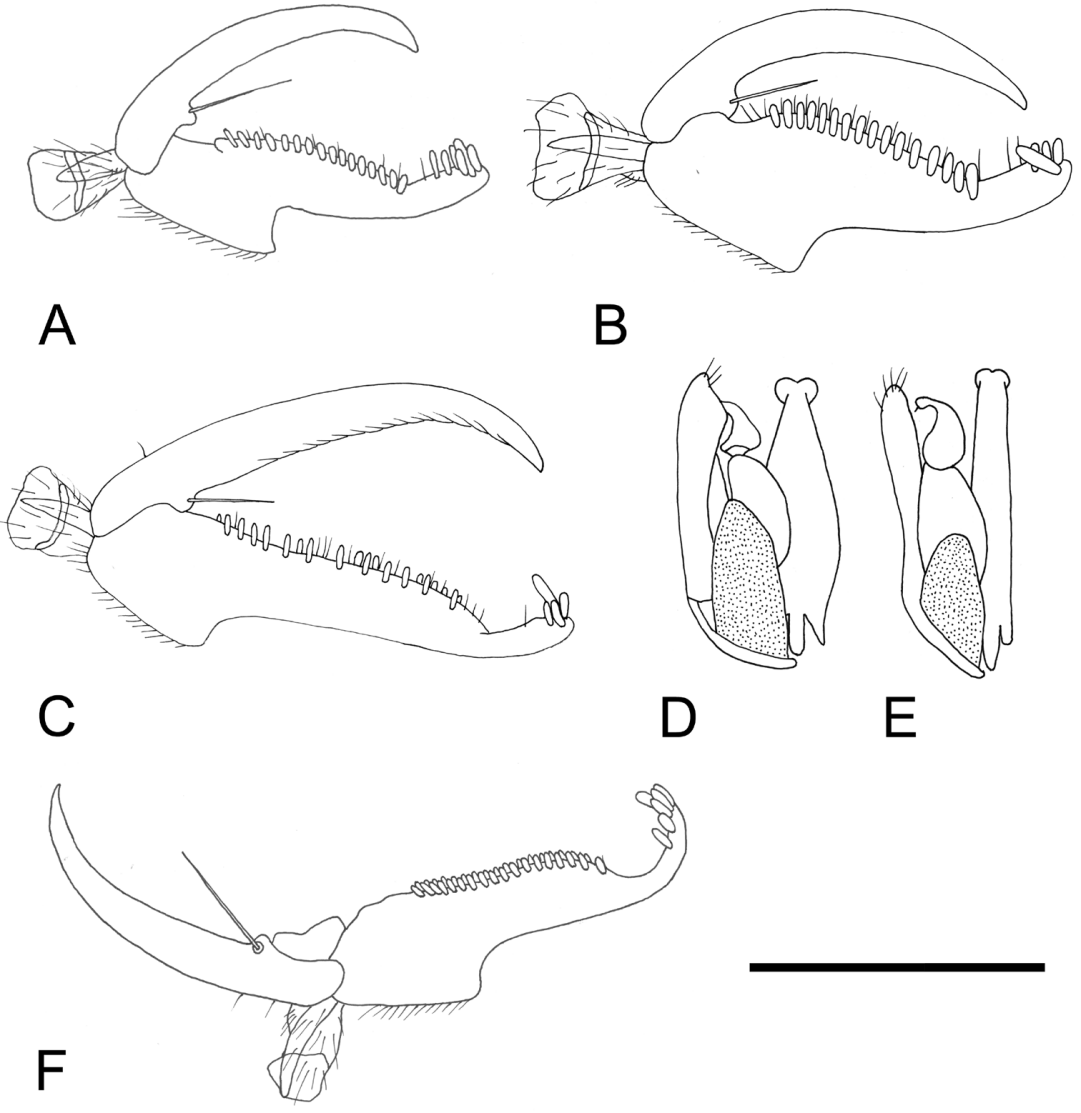
Chela of holotypes: **A** *Anteon dzanganum* **B** *Anteon kibalense* **C** *Anteon mubfs* **D** *Anteon zimbabwense*. Male genitalia of holotypes (right half removed): *Anteon granulatum*; *Anteon makererense*. Scale bar: 0.26 mm (**A**); 0.28 mm (**B**); 0.22 mm (**C**); 0.13 mm (**D, E**); 0.40 mm (**F**).

Female fully winged; head and mesosoma black, except mandible testaceous; head and scutum granulated and reticulate rugose; posterior surface of propodeum reticulate rugose, without longitudinal keels; forewing hyaline, without dark transverse bands or spots, with distal part of stigmal vein less than 0.5 as long as proximal part; segment 4 of protarsus slightly shorter than basal part of segment 5; segment 5 of protarsus (Figs 1A, 4D) with inner side curvilinear, with distinct apical region, basal part shorter than distal part, distal region very short and distal lamellae located near medial lamellae.

####### Description.

***Female*.** Fully winged; length 2.2 mm. Head black, except mandible testaceous; antenna testaceous, except dorsal side of segments 6–10 brownish; metasoma brown; legs testaceous. Antenna clavate; antennal segments in following proportions: 10:5:6:5:4:5:6:5.5:5.5:7. Head dull, granulated and reticulate rugose; frontal line complete; face without two lateral keels around orbits and directed towards antennal toruli; occipital carina complete; POL = 6; OL = 3; OOL = 3: OPL = 4; TL = 4; greatest breadth of posterior ocelli about as long as OL. Pronotum dull, reticulate rugose; posterior surface longer than OPL (7:4) and shorter than scutum (7:14); pronotal tubercle reaching tegula. Scutum dull, granulated and reticulate rugose, except short unsculptured area near anterior margin. Notauli incomplete, reaching about 0.3 length of scutum. Scutellum and metanotum shiny, punctate, unsculptured among punctures. Mesopleuron and metapleuron dull, reticulate rugose. Propodeum dull, with transverse keel between dorsal and posterior surface; dorsal surface reticulate rugose; posterior surface reticulate rugose, without longitudinal keels. Forewing hyaline, without dark transverse bands; distal part of stigmal vein much shorter than proximal part (2.5:9). Protarsal segments in following proportions: 5:2:3:4:13; segment 2 of protarsus produced into hook. Segment 4 of protarsus slightly shorter than basal part of segment 5 (4:6). Segment 5 of protarsus (Figs 1A, 4D) with basal part slightly shorter than distal part (6:7), with inner side curvilinear and with apical region distinct. Enlarged claw (Figs 1A, 4D) with proximal prominence bearing one long bristle. Segment 5 of protarsus (Figs 1A, 4D) with one row of 16 lamellae; distal apex with 6 lamellae. Tibial spurs 1/1/2.

***Male*.** Unknown.

####### Differential diagnosis.

Because of the above diagnosis, the new species is similar to *Anteon canabense* (Benoit, 1951b). The main differences regard the sculpture of the posterior surface of the propodeum and the colour (posterior surface of propodeum granulated, except few irregular keels near margins; head and mesosoma testaceous-reddish, occasionally partly darkened, in *Anteon canabense*; posterior surface of propodeum reticulate rugose; head and mesosoma black, except mandible testaceous, in *Anteon dzanganum*).

####### Etymology.

The species is named after the type locality, Dzanga-Ndoki National Park.

####### Hosts.

Unknown.

####### Distribution.

Central African Republic.

###### Anteon evertsi

Taxon classificationAnimaliaHymenopteraDryinidae

Olmi**

Anteon evertsi Olmi 1989: 159.

####### Material examined.

***New record*. CENTRAL AFRICAN REPUBLIC: *SANGHA-MBAÉRÉ PREFECTURE***: Dzanga-Ndoki National Park, 38.6 km 173°S Lidjombo, 2°21.60'N, 16°03.20'E, 350 m, 23.V.2001, sweep, lowland rainforest, S. van Noort leg., 4♂♂ (SAMC).

####### Hosts.

Unknown.

####### Distribution.

Cameroon, Gambia, Ivory Coast, Kenya (Olmi 1989; Olmi et al. 2015). Newly recorded from Central African Republic here.

###### Anteon fisheri

Taxon classificationAnimaliaHymenopteraDryinidae

Olmi**

Anteon fisheri Olmi, 2003: 24.

####### Material examined.

***New records*. UGANDA: *WESTERN REGION***: Kibale Forest, Kanyawara (RMNH); Kibale National Park, Kanyawara Makerere University Biological Field Station, 0°34.390'S 30°21.658'E, 1587 m, 4–26.VIII.2008, YPT, primary mid-altitude rainforest, near stream, S. van Noort leg., 1♂ (SAMC).

####### Hosts.

Unknown.

####### Distribution.

Madagascar, South Africa (Azevedo et al. 2010; Olmi 2003, 2006). Newly recorded from Uganda here.

###### Anteon granulatum

Taxon classificationAnimaliaHymenopteraDryinidae

sp. n.*

http://zoobank.org/7D0E3446-FDF4-4A96-8537-3CCE51AC202F

Figs 1D , 5

####### Type material.

HOLOTYPE: ♂, **UGANDA: *WESTERN REGION***: Kibale National Park, Kanyawara Makerere University Biological Field Station, 0°33.996'N, 30°21.262'E, 1495 m, 12–18.VIII.2005, UG05-M20, Malaise trap, secondary mid-altitude rainforest, S. van Noort leg. (SAMC).

####### Diagnosis.

Male with clypeus not sculptured by longitudinal and subparallel keels; scutum completely granulated and slightly reticulate rugose; posterior surface of propodeum without longitudinal keels; paramere (Figs 1D, 5F) with small distal inner rounded process and proximal membranous process not provided with mosaic sculpture and sensorial setae; distal inner process of paramere with distal apex situated quite close to distal apex of paramere (Figs 1D, 5F).

####### Description.

***Male*.** Fully winged; length 1.7 mm. Head black, except mandible testaceous; antenna brown; mesosoma black; metasoma brown; legs testaceous. Antenna filiform; antennal segments in following proportions: 8:5:7:7:7:7:7:7:7:8. Head granulated; frontal line complete; face without lateral keels along orbits directed towards antennal toruli; occipital carina complete; POL = 6; OL = 3; OOL = 5; OPL = 3; TL = 4; greatest breadth of posterior ocellus slightly shorter than OPL (2:3). Scutum dull, completely granulated and slightly reticulate rugose. Notauli very short, hardly visible near anterior margin of scutum. Scutellum shiny, slightly granulated. Metanotum shiny, unsculptured. Propodeum completely reticulate rugose, with strong transverse keel between dorsal and posterior surface; posterior surface without longitudinal keels, with areolae about as large as those of dorsal surface. Forewing hyaline, without dark transverse bands or spots; distal part of stigmal vein much shorter than proximal part (1.5:6). Paramere (Figs 1D, 5F) with small distal inner rounded process; proximal membranous process without mosaic sculpture and sensorial setae. Tibial spurs 1/1/2.

***Female*.** Unknown.

####### Differential diagnosis.

Because of the above diagnosis, the new species is similar to *Anteon cautum* Olmi, 1994a, *Anteon emeritum* Olmi, 1984, and *Anteon whartoni* Olmi, in Olmi & Copeland, 2011. The main difference regards the scutum sculpture: completely reticulate rugose and granulated in *Anteon granulatum*; not completely reticulate rugose, at most with small surface near anterior margin rugose in the other three species.

####### Etymology.

The species is named after the head completely granulated.

####### Hosts.

Unknown.

####### Distribution.

Uganda.

###### Anteon gutturnium

Taxon classificationAnimaliaHymenopteraDryinidae

(Benoit)**

Xenanteon gutturnium Benoit, 1951b.
Anteon gutturnium (Benoit): Olmi 1984: 384

####### Material examined.

***Published records*.** Olmi (1984): **UGANDA: *CENTRAL REGION***: Masaka District, Kawanda (BMNH, AMNH); Kampala District, Kampala, V.1938, H.C. Taylor leg., 1♀ (BMNH). ***WESTERN REGION***: Bushenyi District, Bushenyi, III.1939, H.C. Taylor leg., 1♀ (BMNH). ***New records*. CENTRAL AFRICAN REPUBLIC: *SANGHA-MBAÉRÉ PREFECTURE***: Reserve Speciale de Forêt dense de Dzanga-Sangha, 12.7 km 326°NW Bayanga, 3°00.27'N, 16°11.55'E, 420 m, 17.V.2001, sweep, lowland rainforest, S. van Noort leg., 3♂♂ (SAMC). **UGANDA: *WESTERN REGION***: Kibale National Park, Kanyawara Makerere University Biological Field Station, 0°33.836'N, 30°21.700'E, 1523 m, 4–26.VIII.2008, YPT, primary mid-altitude rainforest, S. van Noort leg., 3♂♂ (SAMC); same locality label, 0°33.891'N, 30°21.468'E, 1506 m, 4–26.VIII.2008, YPT, primary mid-altitude rainforest, S. van Noort leg., 2♂♂ (SAMC).

####### Hosts.

Unknown.

####### Distribution.

Afrotropical, recorded from almost all sub-saharian countries, from Senegal to Uganda (Olmi 1984, 2006; Olmi and Copeland 2011; Olmi et al. 2015), in addition to Madagascar (Azevedo et al. 2010). Newly recorded from Central African Republic here.

###### Anteon hoyoi

Taxon classificationAnimaliaHymenopteraDryinidae

Olmi**

Anteon hoyoi Olmi, 1984: 390.

####### Material examined.

***New record*. UGANDA: *WESTERN REGION***: Kasese District, Kibale National Park, Kanyawara, Makerere University Biological Field Station, 00°33.836'N, 30°21.700'E, 1523 m, 6.VIII.2008, sweep, primary mid-altitude rainforest, S. van Noort leg., 1♀ (SAMC).

####### Hosts.

Unknown.

####### Distribution.

Democratic Republic of the Congo (Olmi 1984). Newly recorded from Uganda here.

###### Anteon inflatrix

Taxon classificationAnimaliaHymenopteraDryinidae

Benoit**

Anteon inflatrix Benoit, 1951b: 161.

####### Material examined.

***New record*. CENTRAL AFRICAN REPUBLIC: *Sangha-Mbaéré Prefecture***: Dzanga-Ndoki National Park, Mabéa Bai, 21.4 km 53°NE Bayanga, 3°02.01'N, 16°24.57'E, 510 m, 4.V.2001, sweep, lowland rainforest, marsh clearing, S. van Noort leg., 1♀ (SAMC).

####### Hosts.

Unknown.

####### Distribution.

South Africa (Olmi 1984, 2006). Newly recorded from Central African Republic here.

###### Anteon kawandanum

Taxon classificationAnimaliaHymenopteraDryinidae

Olmi

Anteon kawandanum Olmi, 1984: 374.

####### Material examined.

***Published records*.** Olmi (1984). **UGANDA: *Central Region***: Masaka District, Kawanda, V.1943, T.H.C. Taylor leg., holotype ♀ (BMNH); same locality label, 13.VII.1943, VII.1943, 15.VII.1943, 19.VII.1943, 5 paratypes ♂♂, 1 paratype ♀ (BMNH); same locality label, 1 paratype ♀, 1 paratype ♂ (AMNH). ***EASTERN REGION***: Serere District, Serere, VII.1943, T.H.C. Taylor leg., 10 paratypes ♀♀ (BMNH); same locality label, 1 paratype ♀ (AMNH).

####### Hosts.

Unknown.

####### Distribution.

Afrotropical, recorded from almost all sub-saharian countries, from Gambia to Somalia (Olmi 1984, 2006), in addition to Madagascar (Azevedo et al. 2010).

###### Anteon kibalense

Taxon classificationAnimaliaHymenopteraDryinidae

sp. n.*

http://zoobank.org/134FC47E-817B-4EE2-90EC-1046FC55605F

Figs 1B , 6

####### Type material.

HOLOTYPE : ♀, **UGANDA: *WESTERN REGION***: Kibale National Park, Kanyawara Makerere University Biological Field Station, 0°33.996'S 30°21.262'E, 1495 m, 29–31.VII.2005, UG05-Y06, YPT, secondary mid-altitude rainforest, S. van Noort leg. (SAMC). Paratypes: same locality label as holotype, 1 paratype ♀ (SAMC), 1 paratype ♀ (MOLC).

####### Diagnosis.

Female fully winged; head and mesosoma black, except mandible brown; head completely reticulate rugose; posterior surface of propodeum reticulate rugose, without longitudinal keels, with areolae about as large as those of dorsal surface; forewing with two dark transverse bands, with distal part of stigmal vein less than 0.5 as long as proximal part; segment 4 of protarsus slightly shorter than basal part of segment 5; segment 5 of protarsus (Figs 1B, 6F) with inner side curvilinear, with distinct apical region, with basal part slightly shorter than distal part.

####### Description.

***Female*.** Fully winged; length 2.1–3.3 mm (holotype 2.8 mm). Head black, except mandible brown; antenna brown; mesosoma black; metasoma brown; legs brown, except articulations, trochanters, procoxa and mesocoxa testaceous. Antenna clavate; antennal segments in following proportions: 10:6:7:6:5:6:6:6:6:8. Head slightly convex, dull, completely reticulate rugose; frontal line complete; face without two lateral keels around orbits and directed towards antennal toruli; occipital carina complete; POL = 8; OL = 4; OOL = 6; OPL = 5; TL = 5; greatest breadth of posterior ocelli shorter than OPL (3:5). Pronotum anteriorly crossed by slight transverse impression, dull, with anterior surface transversely striate; posterior surface with anterior half transversely striate and posterior half slightly granulated, not striate; posterior surface shorter than scutum (7:14); pronotal tubercle reaching tegula. Scutum granulated, rugose and irregularly striate. Notauli absent. Scutellum shiny, unsculptured. Metanotum rugose. Propodeum with strong transverse keel between dorsal and posterior surface; dorsal surface dull, reticulate rugose; posterior surface dull, reticulate rugose, without longitudinal keels, with areolae about as large as those of dorsal surface. Forewing with two dark transverse bands; distal part of stigmal vein much shorter than proximal part (4:9). Protarsal segments in following proportions: 8:3:4:6:16. Segment 4 of protarsus slightly shorter than basal part of segment 5 (6:7). Enlarged claw (Figs 1B, 6F) with proximal prominence bearing one long bristle. Segment 5 of protarsus (Figs 1B, 6F) with basal part slightly shorter than distal part (7:9), with one row of 16 lamellae; distal apex bent and with 5 lamellae. Tibial spurs 1/1/2.

***Male*.** Unknown.

####### Differential diagnosis.

Because of the above diagnosis, the new species is similar to *Anteon zimbabwense* Olmi, 2005b. The main difference regards the segment 5 of the protarsus: with smaller lamellae and distal part slender in *Anteon zimbabwense* (Fig. 1F); with lamellae longer and distal part less slender in *Anteon kibalense* (Fig. 1B, 6F).

####### Etymology.

The species is named after the type locality, Kibale National Park.

####### Hosts.

Unknown.

####### Distribution.

Uganda.

###### Anteon kivuanum

Taxon classificationAnimaliaHymenopteraDryinidae

(Benoit)**

Chelogynus kivuanus Benoit, 1951c: 13.
Anteon kivuanum Olmi, 1984: 363.

####### Material examined.

***New records*. CENTRAL AFRICAN REPUBLIC: *Sangha-Mbaéré Prefecture***: Dzanga-Ndoki National Park, Mabéa Bai, 21.4 km 53°NE Bayanga, 3°02.01'N, 16°24.57'E, 510 m, 4.V.2001, sweep, lowland rainforest, marsh clearing, S. van Noort leg., 3♂♂ (SAMC); Dzanga-Ndoki National Park, 38.6 km 173°S Lidjombo, 2°21.60'N, 16°03.20'E, 350 m, 23.V.2001, sweep, lowland rainforest, S. van Noort leg., 5♂♂ (SAMC); Reserve Speciale de Forêt dense de Dzanga-Sangha, 12.7 km 326°NW Bayanga, 3°00.27'N, 16°11.55'E, 420 m, 17.V.2001, sweep, lowland rainforest, S. van Noort leg., 4♂♂ (SAMC). **UGANDA: *WESTERN REGION***: Kibale Forest, Kanyawara (RMNH); Kibale National Park, Kanyawara Makerere University Biological Field Station, 0°34.390'S 30°21.658'E, 1587 m, 4–26.VIII.2008, YPT, primary mid-altitude rainforest, near stream, S. van Noort leg., 1♀ (SAMC); same locality label, 0°33.836'N, 30°21.700'E, 1523 m, 4–26.VIII.2008, YPT, primary mid-altitude rainforest, S. van Noort leg., 2♀♀, 2♂♂ (SAMC); same locality label, 0°33.891'N, 30°21.468'E, 1506 m, 4–26.VIII.2008, YPT, primary mid-altitude rainforest, S. van Noort leg., 1♀, 1♂ (SAMC); same locality label, 0°33.996'S 30°21.262'E, 1495 m, 29–31.VII.2005, YPT, secondary mid-altitude rainforest, S. van Noort leg., 1♀, 1♂ (MOLC); same locality label, 0°33.784'S 30°21.617'E, 1500 m, 12–26.VIII.2008, Malaise trap, primary mid-altitude rainforest, S. van Noort leg., 1♂ (SAMC).

####### Hosts.

Cicadellidae Iassinae (Guglielmino et al. 2013): in South Africa (Western Cape): *Iassomorphus drakensteini* (Naudé).

####### Distribution.

Democratic Republic of the Congo, Kenya, Madagascar, South Africa and Yemen (Azevedo et al. 2010; Olmi 1984, 2006; Olmi and van Harten 2006; Olmi et al. 2015). Newly recorded from Central African Republic and Uganda here.

###### Anteon makererense

Taxon classificationAnimaliaHymenopteraDryinidae

sp. n.*

http://zoobank.org/6B7329B0-3AB5-4CFB-9A8A-BA707C74FD5C

Figs 1E , 7

####### Type material.

HOLOTYPE: ♂, **UGANDA: *WESTERN REGION***: Kibale National Park, Kanyawara Makerere University Biological Field Station, 0°33.408'S 30°22.603'E, 1587 m, 30.VII–5.VIII.2005, UG05-M10, Malaise trap, degraded mid-altitude rainforest, S. van Noort leg. (SAMC).

####### Diagnosis.

Male with head reticulate rugose and granulated; scutum with anterior third reticulate rugose and remaining surface sculptured by many longitudinal subparallel irregular keels; posterior surface of propodeum not provided with longitudinal keels; propodeum with strong transverse keel between dorsal and posterior surface; paramere (Figs 1E, 7F) without distal inner pointed or rounded process, slightly shorter than penis.

####### Description.

***Male*.** Fully winged; length 1.7 mm. Head black, except mandible testaceous; antenna brown, except segment 1 testaceous; mesosoma black; metasoma brown; legs testaceous-dark. Antenna filiform; antennal segments in following proportions: 9:4:4:4:4:4:4:4:4.5:7. Head completely reticulate rugose and granulated; frontal line complete; occipital carina complete; POL = 6; OL = 3; OOL = 4; OPL = 1.5; TL = 2; greatest breadth of posterior ocelli shorter than OL (2:3). Scutum dull, with anterior third reticulate rugose; remaining surface sculptured by many longitudinal subparallel irregular keels. Notauli very short, hardly visible near anterior margin of scutum. Scutellum and metanotum shiny, unsculptured. Propodeum with strong transverse keel between dorsal and posterior surface; dorsal surface reticulate rugose; posterior surface reticulate rugose, sculptured by areolae smaller than those of dorsal surface, without longitudinal keels. Forewing hyaline, without dark transverse bands or spots; distal part of stigmal vein much shorter than proximal part (2:6). Paramere (Figs 1E, 7F) without distal inner pointed process. Tibial spurs 1/1/2.

***Female*.** Unknown.

####### Differential diagnosis.

Because of the above diagnosis, the new species is similar to *Anteon reunionense* Olmi, 1987. The main difference regards the sculpture of the scutum: with anterior half reticulate rugose and remaining surface unsculptured, or slightly granulated in *Anteon reunionense*; with anterior third reticulate rugose and remaining surface sculptured by many longitudinal subparallel irregular keels in *Anteon makererense*.

####### Etymology.

The species is named after Makerere University.

####### Hosts.

Unknown.

####### Distribution.

Uganda.

###### Anteon mubfs

Taxon classificationAnimaliaHymenopteraDryinidae

sp. n.*

http://zoobank.org/B07F8CE4-936A-41C1-9F99-F330182085B4

Figs 1C , 8A–F

####### Type material.

HOLOTYPE: ♀, **UGANDA: *WESTERN REGION***: Kibale National Park, Kanyawara Makerere University Biological Field Station, 0°35.405'S 30°21.646'E, 1484 m, 4–26.VIII.2008, UG08-KF10-Y02, YPT, primary mid-altitude rainforest, near stream, S. van Noort leg. (SAMC).

####### Diagnosis.

Female fully winged; head reticulate rugose, except vertex behind posterior ocelli and temple granulated; posterior surface of pronotum with raised carina on both anterior and lateral margins; posterior surface of propodeum reticulate rugose, without longitudinal keels; forewing with two dark transverse bands and distal part of stigmal vein less than 0.5 as long as proximal part; segment 4 of protarsus approximately longer than basal part of segment 5; segment 5 of protarsus (Figs 1C, 8F) with inner side curvilinear, with distinct apical region, with basal part shorter than distal part.

####### Description.

***Female*.** Fully winged; length 2.6 mm. Head black, except mandible and part of anterior half of clypeus testaceous; antenna testaceous; mesosoma black; metasoma brown; legs testaceous. Antenna clavate; antennal segments in following proportions: 8:6:7:6:5:5:6:6:6:8. Head dull, reticulate rugose, except vertex behind posterior ocelli and temple granulated; frontal line complete, partly continuing also behind the anterior ocellus; face with two lateral keels along orbits and directed towards antennal toruli; occipital carina complete; POL = 6; OL = 4; OOL = 4 OPL = 4; TL = 5; greatest breadth of posterior ocelli shorter than OPL (3:4). Pronotum with slight transverse anterior impression; anterior surface short, transversely striate, hidden behind head; posterior surface slightly shorter than scutum (11:14), shiny, granulated, except few irregular keels near anterior margin; posterior surface with raised carina on anterior and lateral margins; pronotal tubercle reaching tegula. Scutum shiny, slightly granulated, with some irregular keels on lateral surfaces. Notauli absent. Scutellum and metanotum shiny, unsculptured. Propodeum with transverse keel between dorsal and posterior surface; dorsal surface reticulate rugose; posterior surface reticulate rugose, with with areolae about as large as those of dorsal surface, without longitudinal keels. Forewing with two dark transverse bands; distal part of stigmal vein much shorter than proximal part (4:9). Protarsal segments in following proportions: 6:2:3:10:18; protarsal segment 2 produced into hook; protarsal segment 4 much longer than basal part of protarsal segment 5 (10:5). Enlarged claw (Figs 1C, 8F) with a proximal prominence bearing one long bristle. Segment 5 of protarsus (Figs 1C, 8F) with basal part much shorter than distal part (5:13), with two rows of 8+12 lamellae; distal apex with 4 lamellae. Tibial spurs 1/1/2.

***Male*.** Unknown.

####### Differential diagnosis.

Because of the above diagnosis, the new species is similar to *Anteon abditum* Olmi, 1994a. The main difference regards the shape of the pronotum: without raised carinae in *Anteon abditum*; with raised carina on anterior and lateral margins in *Anteon mubfs*.

####### Etymology.

Named after the acronym of Makerere University Biological Field Station, where the holotype was collected. The field station is affectionately called “Mubfs” by those privileged to have experienced a stay there. Noun in apposition.

####### Hosts.

Unknown.

####### Distribution.

Uganda.

###### Anteon ngoyense

Taxon classificationAnimaliaHymenopteraDryinidae

Olmi

Anteon ngoyense Olmi, 2009: 451.

####### Material examined.

***Published record*.** Olmi et al. (2015: **CENTRAL AFRICAN REPUBLIC: *Sangha-Mbaéré Prefecture***: Dzanga-Ndoki National Park, Mabéa Bai,21.4 Km 53°NE Bayanga, 03°02'01"N 16°24'57"E, 510 m, 4–5.V.2001, Malaise trap, lowland rainforest, marsh clearing, S. van Noort leg., 1♂ (SAMC).

####### Hosts.

Unknown.

####### Distribution.

Central African Republic, South Africa, Uganda (Olmi et al. 2015).

###### Anteon semajanna

Taxon classificationAnimaliaHymenopteraDryinidae

Olmi, Copeland & Guglielmino**

Fig. 9

Anteon semajanna Olmi, Copeland & Guglielmino, 2015: 349.

####### Material examined.

***Published record*.** Olmi et al. (2015: **UGANDA: *WESTERN REGION***: Kibale National Park, Kanyawara Makerere University Biological Field Station, 0°33.871'S 30°21.355'E, 1495 m, 12–26.VIII.2008, UG08-KF2-M12, Malaise trap, secondary mid-altitude rainforest, S. van Noort leg., 2 paratypes ♂♂ (SAMC). ***New record*. CENTRAL AFRICAN REPUBLIC: *Sangha-Mbaéré Prefecture***: Dzanga-Ndoki National Park, 38.6 km 173°S Lidjombo, 2°21.60'N, 16°03.20'E, 350 m, 23.V.2001, sweep, lowland rainforest, S. van Noort leg., 2♂♂ (SAMC).

####### Hosts.

Unknown.

####### Distribution.

Kenya, Uganda (Olmi et al. 2015). Newly recorded from Central African Republic here.

###### Anteon striatum

Taxon classificationAnimaliaHymenopteraDryinidae

Olmi

Anteon striatum Olmi, 2005b: 233.

####### Material examined.

***Published record*.** Olmi (2005b): **UGANDA: *CENTRAL REGION***: Mubende District, Mulange, XI.1922, R. Dummer leg., SAM-HYM-PO03772, holotype ♂ (SAMC). ***New record*. SOUTH AFRICA: *KwaZulu-Natal***: Karkloof, 29°19.1'S 30°15.5'E, 1325 m, 25.VII–25.IX.2005, Malaise trap, M. Mostovski leg., 1♂ (NMSA).

####### Hosts.

Unknown.

####### Distribution.

Uganda (Olmi 2005b). Newly recorded from South Africa here.

###### Anteon taylori

Taxon classificationAnimaliaHymenopteraDryinidae

Olmi*

Anteon taylori Olmi, 1984: 366.

####### Material examined.

***Published record*.** Olmi (1984): **UGANDA: *CENTRAL REGION***: Masaka District, Kawanda, XI.1942, T.H.C. Taylor leg., holotype ♀ (BMNH).

####### Hosts.

Unknown.

####### Distribution.

Uganda (Olmi 1984).

###### Anteon townesi

Taxon classificationAnimaliaHymenopteraDryinidae

Olmi**

Anteon townesi Olmi, 1984: 379.

####### Material examined.

***New record*. UGANDA: *WESTERN REGION***: Kibale National Park, Kanyawara Makerere University Biological Field Station, 0°35.442'S 30°21.741'E, 1465 m, 10.VIII.2008, sweep, primary mid-altitude rainforest, near stream, S. van Noort leg., 1♀ (SAMC).

####### Hosts.

Unknown.

####### Distribution.

Democratic Republic of the Congo and South Africa (Olmi 1984, 2006). Newly recorded from Uganda here.

###### Anteon ugandanum

Taxon classificationAnimaliaHymenopteraDryinidae

Olmi

Anteon ugandanum Olmi, 1984: 372.

####### Material examined.

***Published records*.** Olmi (1984): **UGANDA: *CENTRAL REGION***: Masaka District, Kawanda, 16.VII.1943, T.H.C. Taylor leg., holotype ♀ (BMNH); same locality label, 15.VII.1943, VIII.1943, 2 paratypes ♀♀ (BMNH); same locality label, 9 paratypes (5♀♀, 4♂♂) (AMNH). ***EASTERN REGION***: Serere District, Serere, VII.1943, T.H.C. Taylor leg., 6 paratypes ♀♀ (BMNH); Busoga Kingdom, Bussu, 1909, E. Bayon leg., 1♀ (MSNG).

####### Hosts.

Unknown.

####### Distribution.

Afrotropical, recorded from almost all sub-saharian countries, from Senegal and Mali to Kenya and Uganda (Olmi 1984, 2006; Olmi et al. 2015).

###### Anteon whartoni

Taxon classificationAnimaliaHymenopteraDryinidae

Olmi

Anteon whartoni Olmi, in Olmi & Copeland, 2011: 180.

####### Material examined.

***Published records*.** (Olmi et al. 2015): **CENTRAL AFRICAN REPUBLIC: *Sangha-Mbaéré Prefecture***: Reserve Speciale de Forêt dense de Dzanga-Sangha, 12.7 km 326°NW Bayanga, 3°00.27'N, 16°11.55'E, 420 m, 17.V.2001, sweep, lowland rainforest, S. van Noort leg., 6♂♂ (5 in SAMC, 1 in MOLC); Dzanga-Ndoki National Park, Mabéa Bai, 21.4 km 53°NE Bayanga, 3°02.01'N, 16°24.57'E, 510 m, 4.V.2001, sweep, lowland rainforest, marsh clearing, S. van Noort leg., 2♂♂ (SAMC).

####### Hosts.

Unknown.

####### Distribution.

Central African Republic, Kenya (Olmi et al. 2015).

###### Anteon zairense

Taxon classificationAnimaliaHymenopteraDryinidae

Benoit**

Anteon zairense Benoit 1951d: 21.

####### Material examined.

***New records*. CENTRAL AFRICAN REPUBLIC: *Sangha-Mbaéré Prefecture***: Dzanga-Ndoki National Park, 38.6 km 173°S Lidjombo, 2°21.60'N, 16°03.20'E, 350 m, 23.V.2001, sweep, lowland rainforest, S. van Noort leg., 6♂♂ (SAMC). **UGANDA: *WESTERN REGION***: Kibale National Park, Kanyawara Makerere University Biological Field Station, 0°33.784'S 30°21.617'E, 1500 m, 2–12.VIII.2008, Malaise trap, primary mid-altitude rainforest, S. van Noort leg., 1♂ (SAMC).

####### Hosts.

Unknown.

####### Distribution.

Afrotropical, recorded from Gabon to Kenya (Olmi et al. 2015), in addition to Madagascar (Azevedo et al. 2010). Newly recorded from Central African Republic and Uganda here.

#### Subfamily Bocchinae Richards, 1939a

##### Genus *Bocchus* Ashmead

*Bocchus* Ashmead, 1893: 91.

###### Bocchus bini

Taxon classificationAnimaliaHymenopteraDryinidae

Olmi**

Bocchus bini Olmi, 1984: 629.

####### Material examined.

***New record*. UGANDA: *WESTERN REGION***: Ankole, Kichwamba, 23–29.IV.1968, 1♀, 1♂ (USNM); same locality label, 1♀ (AMNH).

####### Hosts.

Unknown.

####### Distribution.

Afrotropical, recorded from Ghana to Somalia and Yemen (Olmi and van Harten 2006; Olmi et al. 2015), in addition to Madagascar (Azevedo et al. 2010. Newly recorded from Uganda here.

###### Bocchus kibalensis

Taxon classificationAnimaliaHymenopteraDryinidae

sp. n.*

http://zoobank.org/A1DB1304-4CB8-4463-B291-9AACB6EE5355

Figs 2A , 10

####### Type material.

HOLOTYPE: ♂, **UGANDA: *WESTERN REGION***: Kibale National Park, Kanyawara, Makerere University Biological Field Station, 0°33.836'N, 30°21.700"E, 1523 m, 12–26.VIII.2008, UG08-KF8-M18, Malaise trap, primary mid-altitude rainforest, S. van Noort leg. (SAMC).

####### Diagnosis.

Male with OPL slightly longer than POL; antennal segment 6 less than twice as long as broad; scutum and scutellum completely reticulate rugose; notauli absent; posterior surface of propodeum with median area crossed by numerous transverse keels.

####### Description.

**Figure 2.**
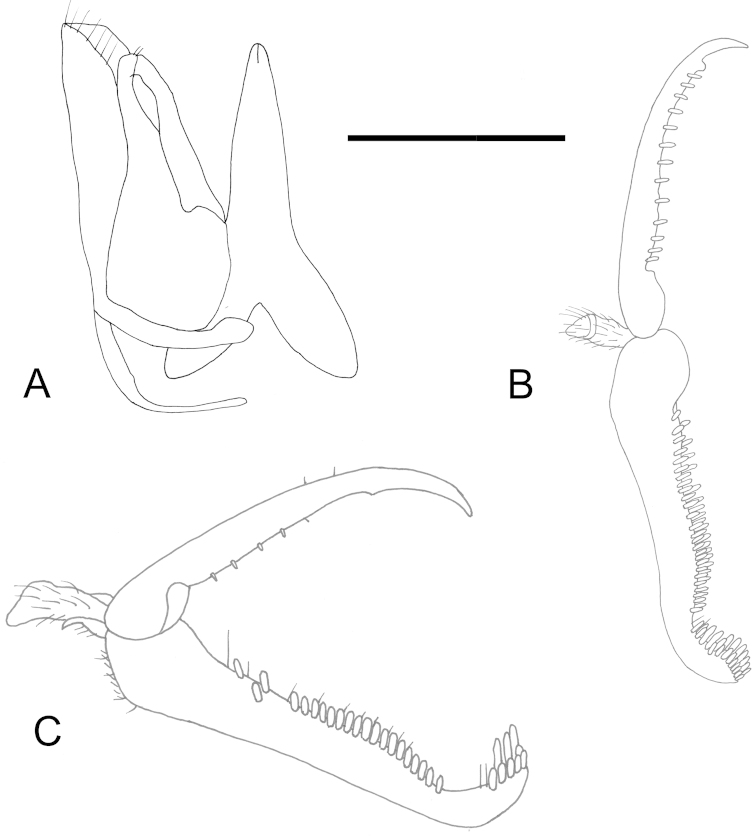
**A** male genitalia of *Bocchus kibalensis* (right half removed) **B, C** chelae of holotypes: **B** *Dryinus kibalus* **C** *Gonatopus kanyawarus*. Scale bar: 0.13 mm (**A**); 0.65 mm (**B**); 0.30 mm (**C**).

***Male*.** Fully winged; length 2.9 mm. Head black, except mandible testaceous; antenna brown, except segments 1–2 ferruginous; mesosoma black; metasoma brown; legs brown, with articulations, tarsi and protibia testaceous. Antenna filiform; antennal segments in following proportions: 14:6:8:8:7:7:7:7:7:10; antennal segment 6 less than twice as long as broad (7:5). Head convex, dull, covered with short hairs, completely reticulate rugose; clypeus reticulate rugose; frontal line complete, with pointed protrusion between antennal toruli; occipital carina complete; POL = 5; OL = 3; OOL = 7; OPL = 7; TL = 7; greatest breadth of posterior ocelli shorter than POL (4:5). Scutum, scutellum and metanotum dull, completely reticulate rugose. Notauli absent. Mesopleuron dull, reticulate rugose. Metapleuron dull, sculptured by numerous strong transverse keels. Propodeum with strong transverse keel between dorsal and posterior surface; dorsal surface dull, reticulate rugose; posterior surface of propodeum with two complete longitudinal keels, median area crossed by many transverse keels and lateral areas completely reticulate rugose. Forewing hyaline, without dark transverse bands; distal part of stigmal vein about as long as proximal part. Genitalia as in Figs 2A, 10F. Tibial spurs 1/1/2.

***Female*.** Unknown.

####### Differential diagnosis.

Because of the above diagnosis, the new species is similar to *Bocchus seyrigi* (Benoit, 1954). The main differences regard OPL/POL ratio (OPL slightly longer than POL in *Bocchus kibalensis*; OPL more than twice as long as POL in *Bocchus seyrigi*) and the sculpture of the median area of posterior surface of propodeum (crossed by many transverse keels in *Bocchus kibalensis*; partly unsculptured in *Bocchus seyrigi*).

####### Etymology.

The species is named after the type locality, in Kibale National Park.

####### Hosts.

Unknown.

####### Distribution.

Uganda.

#### Subfamily Dryininae Haliday, 1833

##### Genus *Dryinus* Latreille

*Dryinus* Latreille, 1804: 176.

###### Dryinus aethiopicus

Taxon classificationAnimaliaHymenopteraDryinidae

(Olmi)

Mesodryinus aethiopicus Olmi, 1984: 1008.
Dryinus aethiopicus (Olmi): Olmi 2004a: 357.

####### Material examined.

***Published record*.** (Olmi 1984; Olmi and Copeland 2011; Olmi et al. 2015): **CENTRAL AFRICAN REPUBLIC: *LOBAYE PREFECTURE***: Boukoko, 1 paratype ♀, 1 paratype ♂ (MNHN).

####### Hosts.

In Central African Republic unidentified Dictyopharidae (Olmi 1984).

####### Distribution.

Central African Republic, Kenya, Sierra Leone (Olmi 1984; Olmi and Copeland 2011).

###### Dryinus erraticus

Taxon classificationAnimaliaHymenopteraDryinidae

(Turner)

Neodryinus erraticus Turner, 1928: 149.
Dryinus erraticus (Turner): Carnegie 1975: 249.

####### Material examined.

***Published records*.** (Olmi et al. 2015): **UGANDA: *WESTERN REGION***: Kibale National Park, Kanyawara, Makerere University Biological Field Station, 0°33.823'N, 30°21.490'E, 1505 m, 4–26.VIII.2008, YPT, primary mid-altitude rainforest, S. van Noort leg., 1♀ (SAMC).

####### Hosts.

Tropiduchidae (Guglielmino et al. 2013): in South Africa and Swaziland: *Numicia viridis* Muir.

####### Distribution.

Afrotropical, recorded from Angola, Democratic Republic of the Congo, Eritrea, Namibia, Somalia, South Africa, Swaziland, Tanzania, Uganda, Zimbabwe (Olmi 1984, 2006, 2009).

###### Dryinus kibalus

Taxon classificationAnimaliaHymenopteraDryinidae

sp. n.*

http://zoobank.org/C8876DFD-C537-442D-AC60-D2C36A488D44

Figs 2B , 11

####### Type material.

HOLOTYPE: ♀, **UGANDA: *WESTERN REGION***: Kibale National Park, Kanyawara, Makerere University Biological Field Station, 0°33.836'N, 30°21.700'E, 1523 m, 4–26.VIII.2008, UG08-KF8-Y07, YPT, primary mid-altitude rainforest, S. van Noort leg., 1♀ (SAMC).

####### Diagnosis.

Female with head black, except mandible and clypeus testaceous; head flat, with posterior margin of vertex convex and TL more than twice as long as POL; head (dorsally viewed) provided with posterior ocelli placed behind imaginary straight line joining posterior edges of eyes; posterior ocelli almost touching occipital carina; head and scutum granulated and reticulate rugose, not sculptured by longitudinal keels or striae; pronotum black, except posterior collar ferruginous; posterior collar of pronotum present; propodeum black; segment 1 of protarsus slightly longer than segment 4; enlarged claw (Figs 2B, 11F) approximately as long as segment 5 of protarsus.

####### Description.

***Female*.** Fully winged; length 7.3 mm. Head black, except mandible and clypeus testaceous; antenna brown, except segments 1–2 and 10 testaceous, distal extremity of segment 4 and proximal third of segment 5 whitish; propleuron brown-black; pronotum black, except posterior collar ferruginous; rest of mesosoma black; metasoma brown-testaceous; legs testaceous. Antenna clavate; antennal segments in following proportions: 12:6:56:33:21:14:10:7:8:10; rhinaria present in segments 6-10. Head dull, granulated and reticulate rugose, without longitudinal keels; occipital carina complete; posterior ocelli almost touching occipital carina, situated just behind virtual straight line joining posterior edges of eyes; POL = 2; OL = 1.5; OOL = 12; OPL = 0.3; TL = 6; greatest breadth of posterior ocelli longer than POL (3:2). Pronotum dull, granulated, with numerous keels around disc, on lateral regions and anterior collar; pronotum crossed by slight anterior transverse impresion and strong posterior transverse furrow; anterior collar little distinct; posterior collar long; pronotal tubercle not reaching tegula. Scutum granulated and reticulate rugose, not sculptured by longitudinal keels. Notauli incomplete, reaching about 0.8 length of scutum. Scutellum granulated, weakly rugose. Metanotum rugose. Propodeum reticulate rugose, without longitudinal keels on posterior surface; dorsal surface much longer than posterior surface. Forewing with two dark transverse bands; distal part of stigmal vein much longer than proximal part (22:8). Protarsal segments in following proportions: 27:5:9:26:41. Segment 3 of protarsus produced into hook. Enlarged claw (Figs 2B, 11F) with one large subdistal tooth and one row of 13 lamellae. Segment 5 of protarsus (Figs 2B, 11F) with two rows of about 48 lamellae; distal apex with approximately 40 lamellae. Tibial spurs 1/1/2.

***Male*.** Unknown.

####### Differential diagnosis.

Because of the above diagnosis, the new species is similar to *Dryinus undulatus* (Benoit, 1950b). The main difference regards the sculpture of the head and scutum: granulated and reticulate rugose, not sculptured by longitudinal keels or striae, in *Dryinus kibalus*; head granulated and with some longitudinal keels and scutum granulated and sculptured by many subparallel longitudinal keels in *Dryinus undulatus*.

####### Etymology.

The species is named after the type locality, Kibale National Park.

####### Hosts.

Unknown.

####### Distribution.

Uganda.

###### Dryinus saussurei

Taxon classificationAnimaliaHymenopteraDryinidae

(Ceballos)**

Lestodryinus saussurei Ceballos, 1936: 48.
Dryinus saussurei (Ceballos): Benoit 1954: 412.

####### Material examined.

***New record*. UGANDA: *WESTERN REGION***: Kibale National Park, Kanyawara, Makerere University Biological Field Station, 0°33.836'N, 30°21.700'E, 1523 m, 4–26.VIII.2008, YPT, primary mid-altitude rainforest, near stream, S. van Noort leg., 2♀♀ (SAMC).

####### Hosts.

Unknown.

####### Distribution.

Madagascar (Olmi 1984, 1994a), South Africa (Olmi 2006), Uganda. Newly recorded from Uganda here.

###### Dryinus shimbanus

Taxon classificationAnimaliaHymenopteraDryinidae

Olmi

Dryinus shimbanus Olmi, in Olmi & Copeland, 2011: 184.

####### Material examined.

***Published record*.** (Olmi et al. 2015): **CENTRAL AFRICAN REPUBLIC: *Sangha-Mbaéré Prefecture***: Dzanga-Sangha Dense Forest Special Reserve, 12.7 km 326°NW Bayanga, 03°00'27"N 16°11'55"E, 420 m, 14–15.V.2001, Malaise trap, lowland rainforest, S. van Noort leg., 1♀ (SAMC).

####### Hosts.

Unknown.

####### Distribution.

Kenya, Central African Republic (Olmi et al. 2015).

###### Dryinus turneri

Taxon classificationAnimaliaHymenopteraDryinidae

Olmi

Lestodryinus ampuliciformis Turner, 1928: 148 (preoccupied).
Dryinus turneri Olmi, Copeland & Guglielmino, 2015 (new name): 359.

####### Material examined.

***Published record*.** (Olmi 1984): **UGANDA: *CENTRAL REGION***: Kampala District, Kampala, 1♀ (AEIC).

####### Hosts.

Unknown.

####### Distribution.

Recorded from many Afrotropical countries, from Nigeria to Somalia (Olmi and Copeland 2011).

###### Dryinus ugandanus

Taxon classificationAnimaliaHymenopteraDryinidae

(Olmi)

Tridryinus ugandanus Olmi, 1984: 935.
Dryinus ugandanus (Olmi): Olmi 2006: 43.

####### Material examined.

***Published record*.** (Olmi 1984): **UGANDA: *CENTRAL REGION***: Kampala District, Kampala, ♀ holotype (AEIC).

####### Hosts.

Unknown.

####### Distribution.

Recorded from Uganda, Sierra Leone and South Africa (Olmi 1984, 2006, 2009).

###### Dryinus undulatus

Taxon classificationAnimaliaHymenopteraDryinidae

(Benoit)

Lestodryinus undulatus Benoit, 1950b: 226.
Dryinus undulatus (Benoit): Olmi 2004a: 357.

####### Material examined.

***Published records*.** (Olmi 1984; Olmi et al. 2015): **CENTRAL AFRICAN REPUBLIC: *Lobaye Prefecture***: 150 km NWW Mbaiki, 04°03'N, 17°02'E, 620 m, 14.VI.2009, J. Halada leg., 1♀ (OLL). **UGANDA: *CENTRAL REGION***: Kampala District, Kampala, IV.1936, T.H.C. Taylor leg., 1♀ (BMNH).

####### Hosts.

Lophopidae (Guglielmino et al. 2013): in Mozambique: *Elasmoscelis cimicoides* Spinola.

####### Distribution.

Afrotropical, recorded from Burundi, Central African Republic, Democratic Republic of the Congo, Kenya, Mozambique and Uganda (Olmi et al. 2015).

##### Genus *Pseudodryinus* Olmi

*Pseudodryinus* Olmi, 1991: 365.

###### Pseudodryinus townesi

Taxon classificationAnimaliaHymenopteraDryinidae

(Olmi)**

Thaumatodryinus townesi Olmi, 1984: 692.
Pseudodryinus townesi (Olmi): Olmi 1991: 369.

####### Material examined.

***Published records*.** (Olmi 1984, 1991): **UGANDA: *CENTRAL REGION***: Kampala District, Kampala, ♀ holotype (AEIC); same locality label as holotype, 1 ♀ paratype (AMNH). ***New record*: CENTRAL AFRICAN REPUBLIC: *Sangha-Mbaéré Prefecture***: Dzanga-Ndoki National Park, Mabéa Bai, 21.4 km 53°NE Bayanga, 3°02.01'N, 16°24.57'E, 510 m, 6.V.2001, sweep, lowland rainforest, marsh clearing, CAR01-S48, S. van Noort leg., 1♀ (SAMC).

####### Hosts.

Unknown.

####### Distribution.

Recorded from South Africa and Uganda (Olmi 1984). Newly recorded from Central African Republic here.

#### Subfamily Gonatopodinae Kieffer, in Kieffer & Marshall, 1906

##### Genus *Echthrodelphax* Perkins

*Echthrodelphax* Perkins, 1903: 36.

###### Echthrodelphax migratorius

Taxon classificationAnimaliaHymenopteraDryinidae

Benoit**

Echthrodelphax migratorius Benoit, 1954: 397.

####### Material examined.

***New record*. UGANDA: *CENTRAL REGION***: Masaka District, Kawanda, X.1942, T. H. C. Taylor leg., 1♂ (BMNH).

####### Hosts.

Delphacidae Delphacinae (Guglielmino et al. 2013): in Egypt: nymphs of *Sogatella furcifera* (Horváth) and *Sogatella vibix* (Haupt); in Mozambique: *Sogatella petax* Fennah.

####### Distribution.

Recorded from many Afrotropical countries, from the Cape Verde Islands, Niger and Senegal to Madagascar and Somalia, in addition to Oman and Egypt (Olmi 1984, 1994b, 2006; Olmi et al. 2015). Newly recorded from Uganda here.

###### Echthrodelphax tauricus

Taxon classificationAnimaliaHymenopteraDryinidae

Ponomarenko**

Echthrodelphax tauricus Ponomarenko, 1970: 432.

####### Material examined.

***New record*. CENTRAL AFRICAN REPUBLIC: *Sangha-Mbaéré Prefecture***: Dzanga-Ndoki National Park, Mabéa Bai, 21.4 km 53°NE Bayanga, 3°02.01'N, 16°24.57'E, 510 m, 7.V.2001, sweep, lowland rainforest, marsh clearing, S. van Noort leg., 19♂♂ (18 in SAMC, 1 in MOLC).

####### Hosts.

Delphacidae Delphacinae (Guglielmino et al. 2013): in the Afrotropical region: in the Cape Verde Islands: *Peregrinus maidis* (Ashmead); in Mozambique: *Nycheuma endymion* (Fennah), *Metadelphax propinqua* (Fieber).

####### Distribution.

Recorded from many Afrotropical countries (from the Cape Verde Islands to Somalia, including Madagascar and South Africa), in addition to many European countries, Oman, Yemen and the United Arab Emirates (Azevedo et al. 2010; Olmi 1999b, 2004c, 2005c, 2008; Olmi and van Harten 2000, 2006). Newly recorded from Central African Republic here.

##### Genus *Adryinus* Olmi

*Adryinus* Olmi, 1984: 1126.

###### Adryinus bellicosus

Taxon classificationAnimaliaHymenopteraDryinidae

(Benoit)

Neodryinus bellicosus Benoit, 1950b: 227.
Adryinus bellicosus (Benoit): Olmi 1984: 1132.

####### Material examined.

***Published records*.** Olmi (1984): **UGANDA: *CENTRAL REGION***: Mubende District, Namutamba, 2♀♀ (BMNH, AMNH); ***WESTERN REGION***: Rukungiri District, Rujumbura County, Ruzumbura (= Rujumbura; Ruzhumbura; Ruzumbusa, misspellings in Olmi 1984) [00°40’ 00” S, 029°52’ 00” E], V.1939, T.H.C. Taylor leg., 1♀ (BMNH); ***New record*. UGANDA: *WESTERN REGION***: Masindi District, Budongo Forest near Sonso, 01°45'N, 31°35'E, VI.1995, Th. Wagner leg., 1♀ (CNCI).

####### Hosts.

Unknown.

####### Distribution.

Recorded from Democratic Republic of the Congo, Uganda and Zimbabwe (Olmi 1984).

###### Adryinus oweni

Taxon classificationAnimaliaHymenopteraDryinidae

Olmi

Adryinus oweni Olmi, 1984: 1135.

####### Material examined.

***Published record*.** Olmi (1984): **UGANDA: *CENTRAL REGION***: Kampala District, Kampala, ♀ holotype (AEIC). ***New record*. IVORY COAST: *Bouaké Department***: Bouaké, 11.X.1980, Pitfall trap, 1♀ (AMNH).

####### Hosts.

Unknown.

####### Distribution.

Uganda (Olmi 1984). Newly recorded from Ivory Coast here.

##### Genus *Gonatopus* Ljungh

*Gonatopus* Ljungh, 1810: 161.

###### Gonatopus nearcticus

Taxon classificationAnimaliaHymenopteraDryinidae

(Fenton)

Pachygonatopus nearcticus Fenton, 1927: 6.
Platygonatopus ugandanus Benoit, 1951a: 300 (synonymized by Olmi 1993).
Gonatopus nearcticus (Fenton): Olmi 1993: 80.

####### Material examined.

***Published records*.** Olmi (1984): **UGANDA: *EASTERN REGION***: Busiki (=Namutumba) District, Bululo (= actually Bulule ?), 1909, E. Bayon leg., ♀ holotype of *Platygonatopus ugandanus* (MSNG); Serere District, Serere, VII.1943, T.H.C. Taylor leg., 1♀ (BMNH). ***CENTRAL REGION***: Masaka District, Kawanda, V.1943, VI.1943, T.H.C. Taylor leg., 3♀♀, 3♂♂ (BMNH); same locality label, 1♀, 1♂ (AMNH).

####### Hosts.

Cicadellidae (Guglielmino et al. 2013): in the Afrotropical region: in Namibia: *Paradorydium spatulatum* (Naudé); in South Africa: *Balclutha frontalis* (Ferrari) (= *Balclutha rosea* (Scott)).

####### Distribution.

Recorded from many countries of the Palaearctic, Afrotropical and Nearctic Regions (Olmi et al. 2015); in Africa recorded from many sub-saharian countries, from Benin to South Africa and Ethiopia (Olmi 1984, 2006).

###### Gonatopus camerounensis

Taxon classificationAnimaliaHymenopteraDryinidae

Olmi**

Gonatopus camerounensis Olmi, 2011: 64.

####### Material examined.

***New record*. CENTRAL AFRICAN REPUBLIC: *Sangha-Mbaéré Prefecture***: Dzanga-Ndoki National Park, Mabéa Bai,21.4 km 53°NE Bayanga, 03°02'01"N 16°24'57"E, 510 m, 6–7.V.2001, Malaise trap, lowland rainforest, marsh clearing, S. van Noort leg., 1♀ (SAMC).

####### Hosts.

Unknown.

####### Distribution.

Cameroon (Olmi 2011). Newly recorded from Central African Republic here.

###### Gonatopus guigliae

Taxon classificationAnimaliaHymenopteraDryinidae

(Benoit)

Digonatopus guigliae Benoit, 1951a: 298.
Gonatopus guigliae (Benoit): Olmi 1984: 1596.

####### Material examined.

***Published record*.** Benoit (1951a): **UGANDA: *EASTERN REGION***: Busoga Kingdom, Bussu, 1910, E. Bayon leg., ♀ holoype (MSNG); same locality label as holotype, 1 ♀ paratype (MRAC) (collecting data have been wrongly reported as “Bussu - 1909” in the original description of Benoit (1951) (Penati & Olmi 2011)).

####### Hosts.

Cicadellidae (Guglielmino et al. 2013): in Mozambique: *Exitianus zuluensis* Ross ; in South Africa: *Exitianus natalensis* Ross and *Exitianus taeniaticeps* (Kirschbaum).

####### Distribution.

Recorded from Mozambique, South Africa and Uganda (Olmi 1984, 1998b, 2006).

###### Gonatopus hyalinus

Taxon classificationAnimaliaHymenopteraDryinidae

Olmi

Gonatopus hyalinus Olmi, 1984: 1607.

####### Material examined.

***Published record*.** Olmi et al. (2015): **UGANDA: *CENTRAL REGION***: Masaka District, Kawanda, VI.1943, T. H. C. Taylor leg., 1♀ (BMNH).

####### Hosts.

Unknown.

####### Distribution.

Recorded from Kenya, South Africa and Uganda (Olmi 1984, 2006; Olmi et al. 2015).

###### Gonatopus incognitus

Taxon classificationAnimaliaHymenopteraDryinidae

Olmi

Gonatopus incognitus Olmi, 1984: 1613.

####### Material examined.

***Published record*.** Olmi (1984): **UGANDA: *CENTRAL REGION***: Masaka District, Kawanda, 7.VII.1943, T.H.C. Taylor leg., ♀ holotype (BMNH).

####### Hosts.

Cicadellidae (Guglielmino et al. 2013): in Burkina Faso: *Cicadulina similis* China; in Democratic Republic of the Congo: *Cicadulina mbila* (Naudé); in Nigeria: *Cicadulina storeyi* China; in South Africa: *Exitianus taeniaticeps* (Kirschbaum); in Tanzania: *Cicadulina bipunctata* (Melichar).

####### Distribution.

Afrotropical, broadly spread from the Cape Verde Islands and Burkina Faso to Uganda, in addition to Madagascar, Yemen and Oman (Azevedo et al. 2010; Olmi 1984, 1994b, 1994a, 2006; Olmi and van Harten 2000).

###### Gonatopus kanyawarus

Taxon classificationAnimaliaHymenopteraDryinidae

sp. n.*

http://zoobank.org/3DC58361-799F-4D33-BEBC-19557810BB35

**Figure 3.**
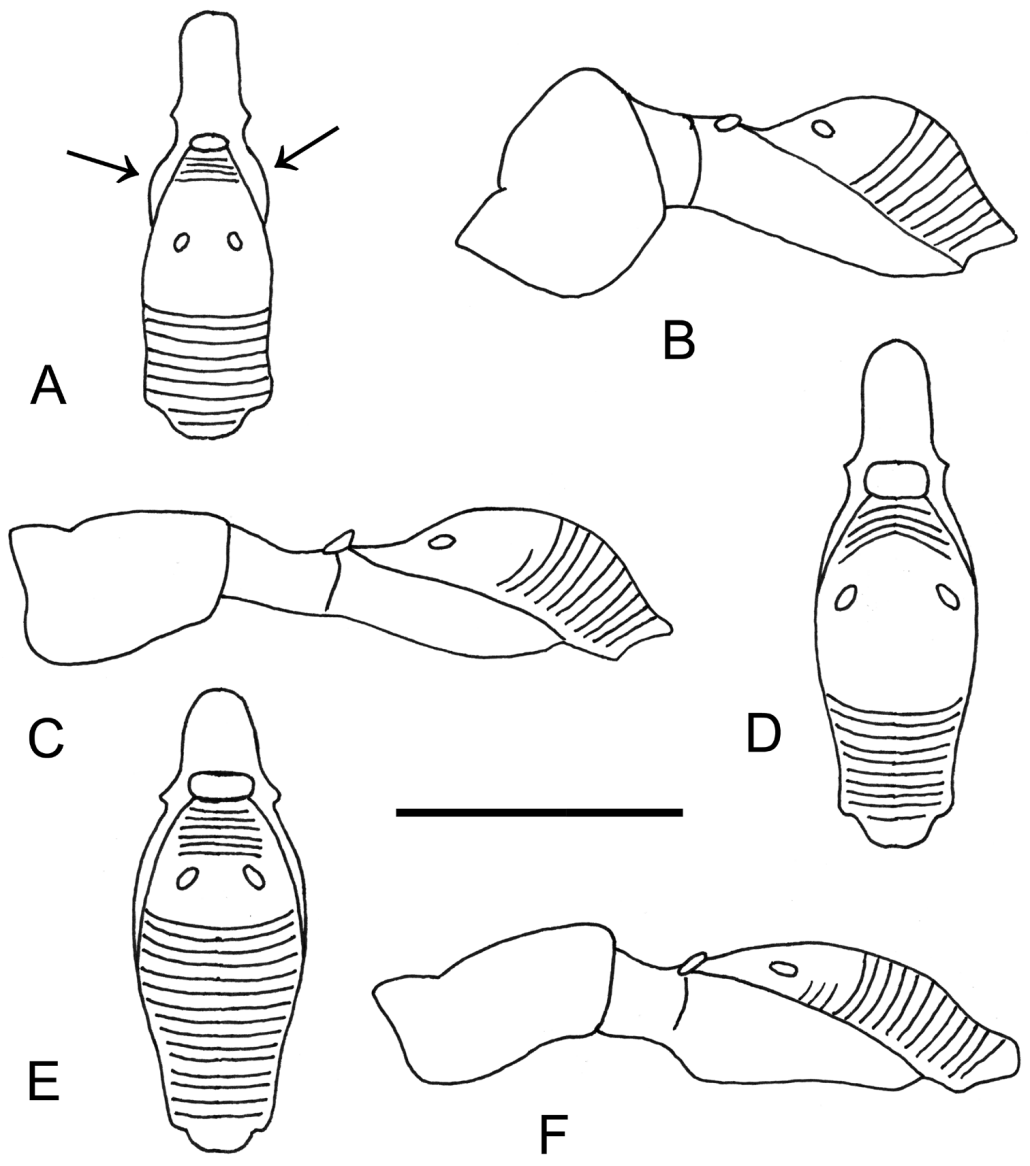
Mesosoma in dorsal (prothorax removed) and lateral view of holotypes: **A, B** *Gonatopus kanyawarus* (rounded protrusions of metanotum indicated by arrows) **C, D** *Gonatopus trochantericus* **E, F** *Gonatopus tulearensis*. Scale bar: 0.87 mm (**A, C, F**); 1.03 mm (**B**); 0.74 mm (**D**); 0.80 mm (**E**).

Figs 2C , 3A , 3B , 12

####### Type material.

HOLOTYPE : ♀, **UGANDA: *WESTERN REGION***: Kibale National Park, Kanyawara, Makerere University Biological Field Station, 0°33.871'N, 30°21.355'E, 1495 m, 4–26.VIII.2008, UG08-KF2-Y03, YPT, primary mid-altitude rainforest, S. van Noort leg. (SAMC). Paratype: same locality label as holotype, 1♀ (SAMC).

####### Diagnosis.

Female with temples without sharp carina; mesosoma black, except posterior third of scutum yellow; scutum less than twice as long as broad, with two lateral pointed apophyses; metanotum very hollow behind scutellum (Fig. 3B), with sides protruding (protrusions rounded) (Fig. 3A); meso-metapleural suture distinct and complete; mesopleuron without lateral pointed prominence; metathorax + propodeum without strong median furrow, shiny, with anterior surface sculptured by numerous longitudinal striae, unsculptured among striae; segment 1 of protarsus shorter than segment 4; subapical tooth of enlarged claw situated very far from distal apex (Figs 2C, 12F).

**Figure 4.**
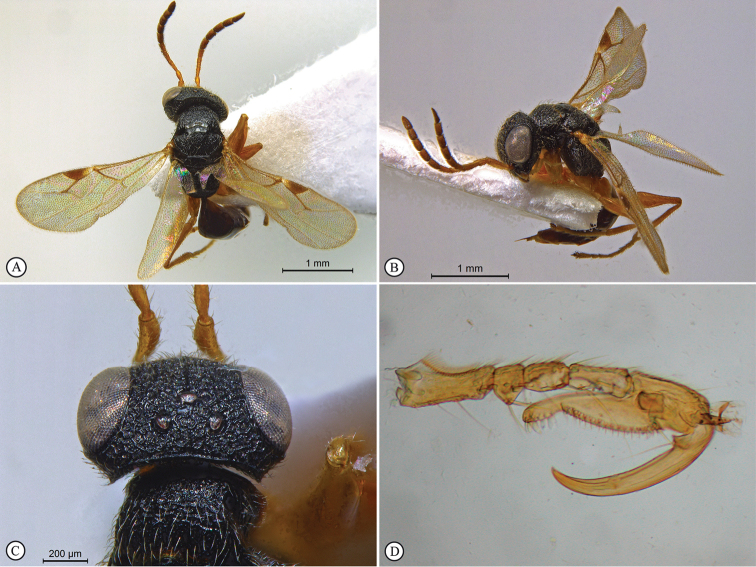
*Anteon dzanganum* sp. n. Holotype female. **A** habitus, dorsal view **B** habitus, lateral view **C** head, pronotum dorsal view **D** chela (slide mounted).

**Figure 5.**
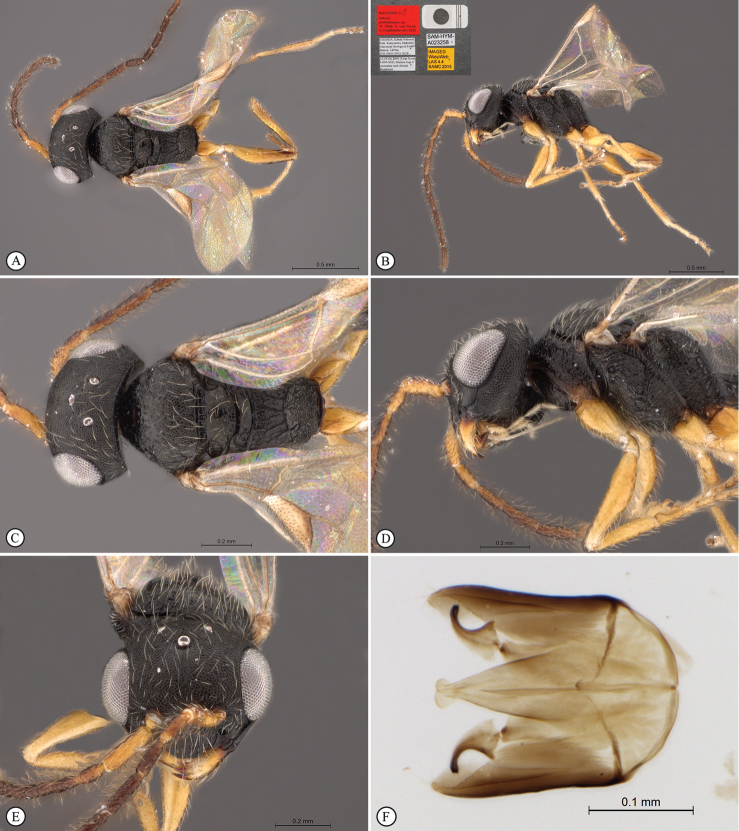
*Anteon granulatum* sp. n. Holotype male. **A** habitus, dorsal view **B** habitus lateral view (inset: data labels) **C** head, mesosoma dorsal view **D** head, mesosoma lateral view **E** head, anterior view **F** male genitalia (slide mounted).

**Figure 6.**
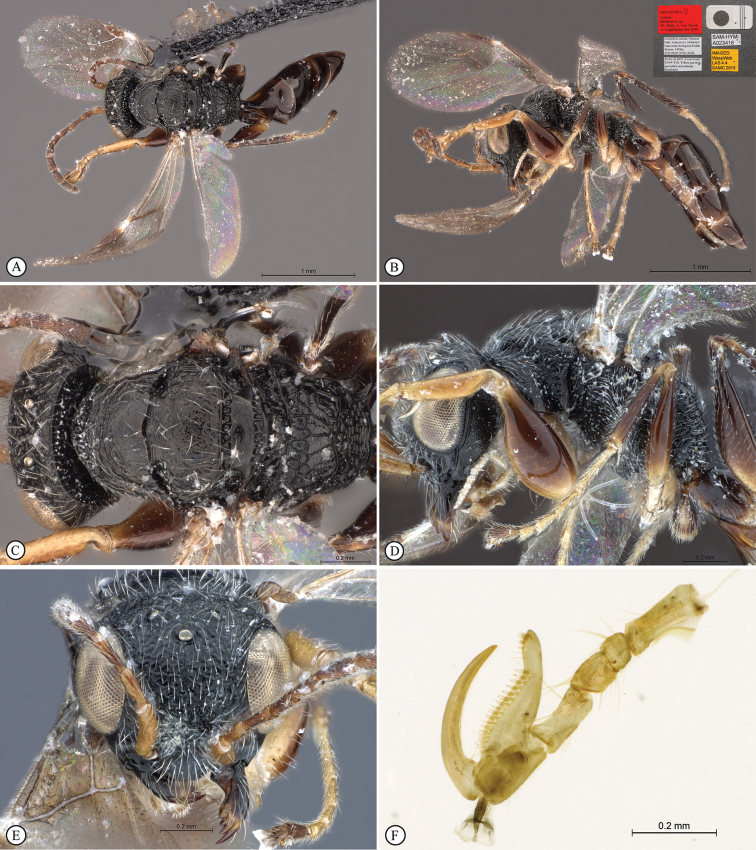
*Anteon kibalense* sp. n. Holotype female. **A** habitus, dorsal view **B** habitus lateral view (inset: data labels) **C** head, mesosoma dorsal view **D** head, mesosoma lateral view **E** head, anterior view **F** chela (slide mounted).

**Figure 7.**
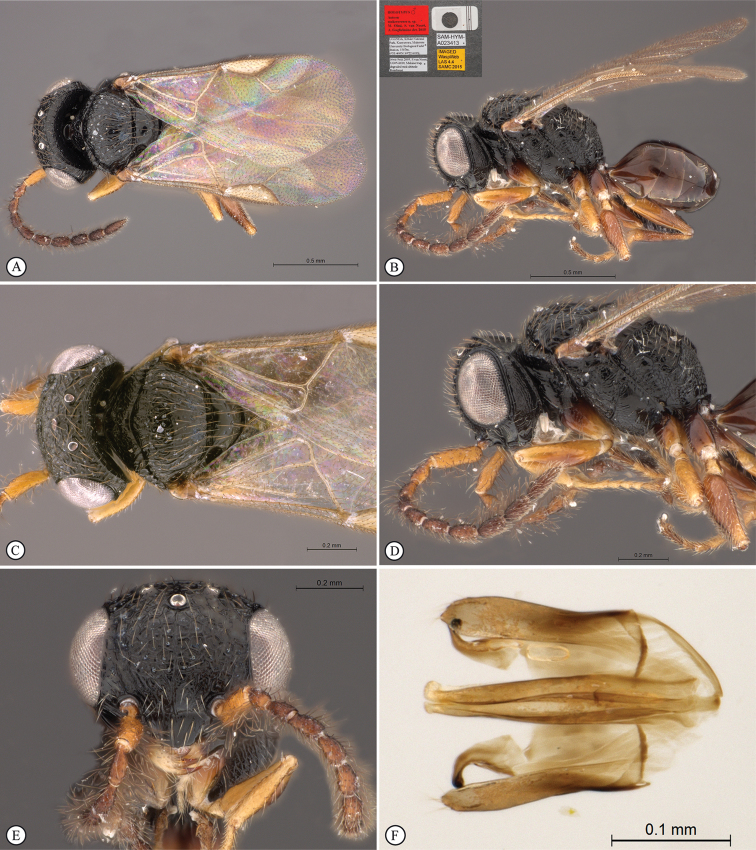
*Anteon makererense* sp. n. Holotype male. **A** habitus, dorsal view **B** habitus lateral view (inset: data labels) **C** head, mesosoma dorsal view **D** head, mesosoma lateral view **E** head, anterior view **F** male genitalia (slide mounted).

**Figure 8.**
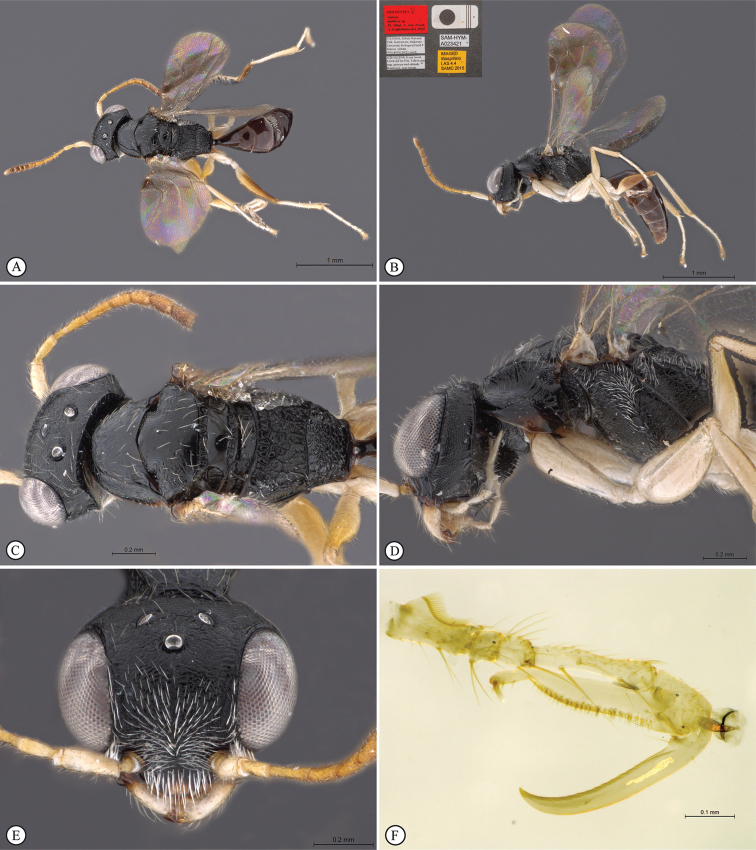
*Anteon mubfs* sp. n. Holotype female. **A** habitus, dorsal view **B** habitus lateral view (inset: data labels) **C** head, mesosoma dorsal view **D** head, mesosoma lateral view **E** head, anterior view **F** chela (slide mounted).

**Figure 9.**
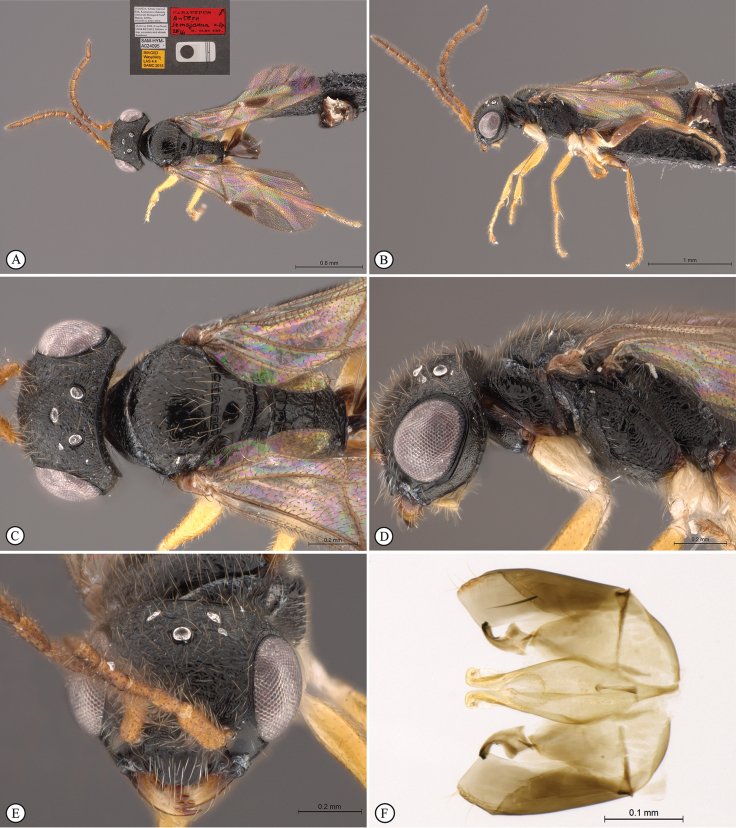
*Anteon semajanna* Olmi, Copeland & Guglielmino, 2015. Paratype male from Uganda, Kibale National Park. **A** habitus, dorsal view **B** habitus lateral view (inset: data labels) **C** head, mesosoma dorsal view **D** head, mesosoma lateral view **E** head, anterior view **F** male genitalia (slide mounted).

**Figure 10.**
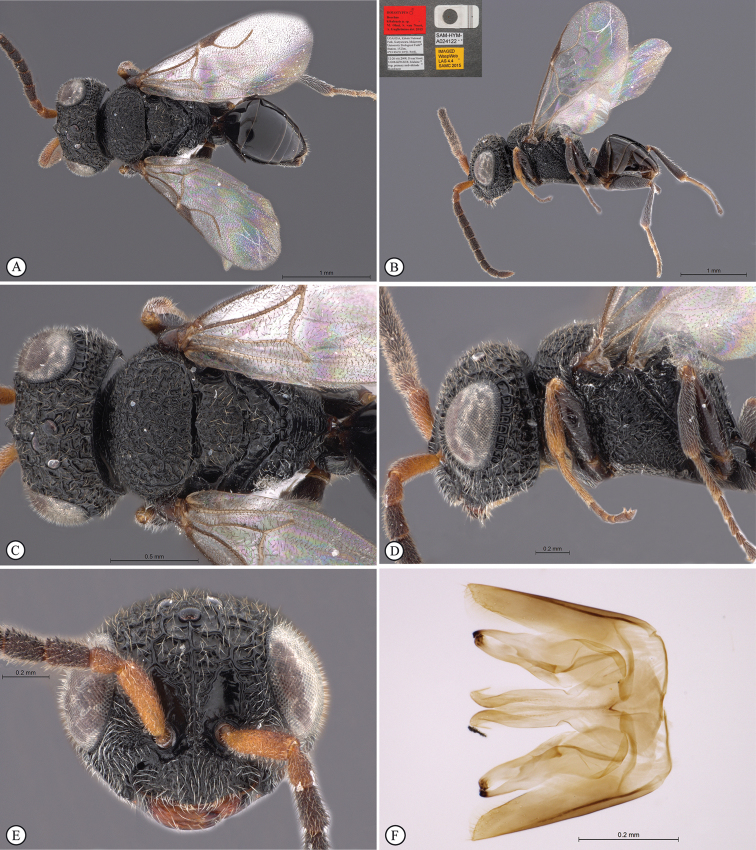
*Bocchus kibalensis* sp. n. Holotype male. **A** habitus, dorsal view **B** habitus lateral view (inset: data labels) **C** head, mesosoma dorsal view **D** head, mesosoma lateral view **E** head, anterior view **F** male genitalia (slide mounted).

**Figure 11.**
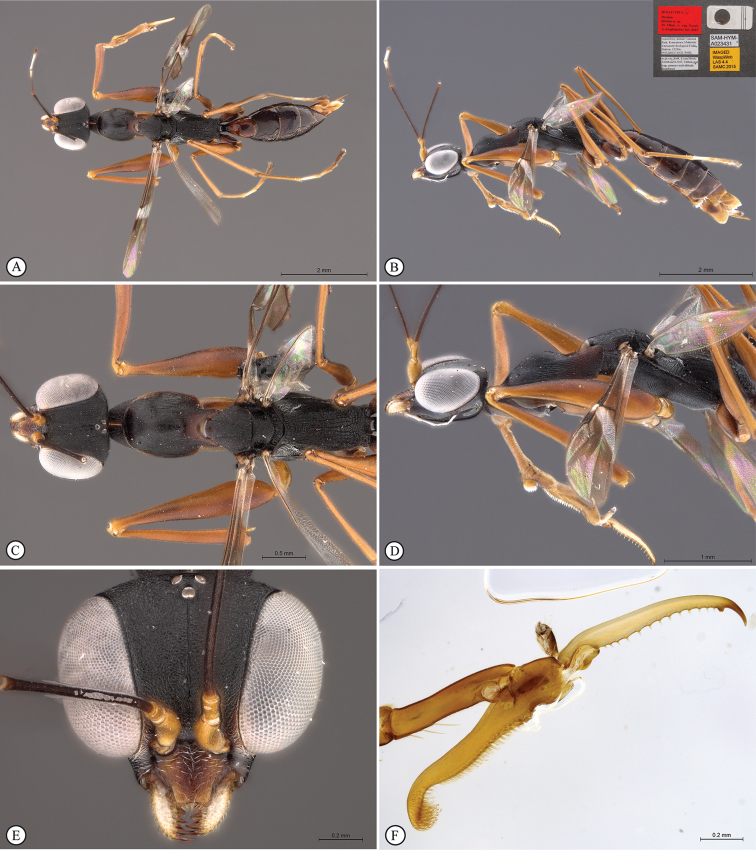
*Dryinus kibalus* sp. n. Holotype female. **A** habitus, dorsal view **B** habitus lateral view (inset: data labels) **C** head, mesosoma dorsal view **D** head, mesosoma lateral view **E** head, anterior view **F** chela (slide mounted).

**Figure 12.**
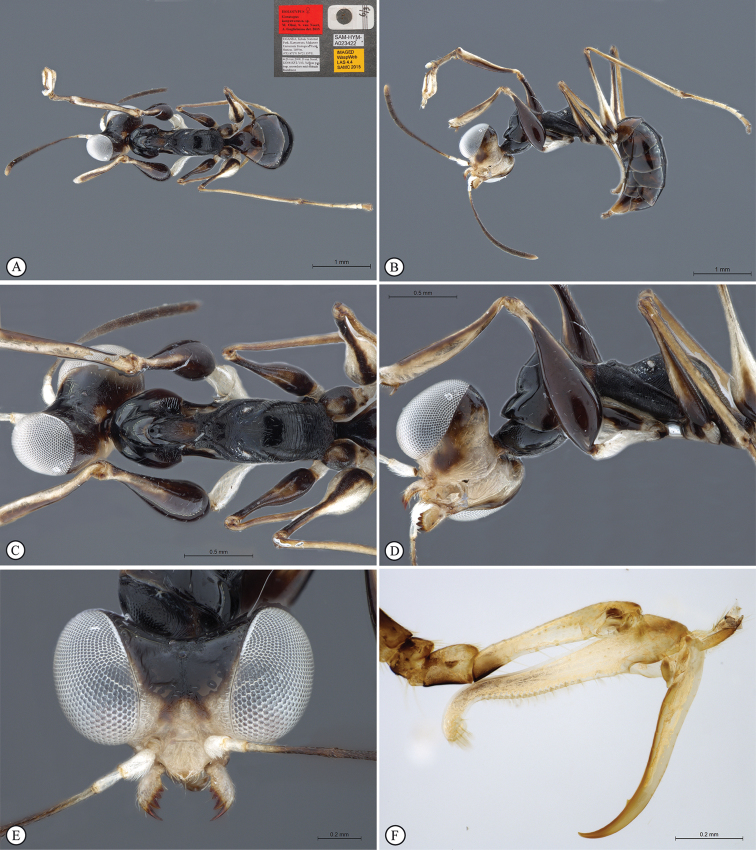
*Gonatopus kanyawarus* sp. n. Holotype female. **A** habitus, dorsal view **B** habitus lateral view (inset: data labels) **C** head, mesosoma dorsal view **D** head, mesosoma lateral view **E** head, anterior view **F** chela (slide mounted).

####### Description.

***Female*.** Apterous; length 3.5–4.0 mm. Head brown-black, except mandible, clypeus, anterior half of face (with short stripe along orbits) and ventral side whitish; antenna brown, except segments 1–2 and proximal third of 3 whitish, segment 10 testaceous; mesosoma black, except posterior third of scutum yellow; metasoma brown-testaceous; fore leg brown, except part of coxa, trochanter, stalk of femur, part of tibiae and part of chela testaceous; mid leg brown, except part of coxa, trochanter, part of femur and tibia, tarsus testaceous; hind leg brown, except distal extremity of coxa, trochanter, part of femur, tibia and tarsus testaceous. Antenna clavate; antennal segments in following proportions: 9:6:21:12:9:9:7:6:5:9. Head excavated, shiny, unsculptured; frontal line incomplete, absent in anterior third of face; occipital carina absent; POL = 2.5; OL = 2; OOL = 10. Palpal formula: 6/3. Pronotum crossed by strong transverse furrow, shiny, unsculptured. Scutum shiny, sculptured by numerous longitudinal striae, laterally with two strong pointed apophyses situated on sides of scutellum. Scutellum shiny, smooth, inclined. Metanotum very long, transversely striate and hollow behind scutellum (Fig. 3B), with sides protruding (protrusions rounded) (Fig. 3A). Metathorax + propodeum shiny, with anterior surface sculptured by numerous longitudinal striae, unsculptured among striae; posterior surface of propodeum, mesopleuron and metapleuron transversely striate. Meso-metapleural suture distinct and complete. Protarsal segments in following proportions: 17:3:6:20:31. Segment 3 of protarsus produced into hook. Enlarged claw (Figs 2C, 12F) with one small subapical tooth and one row of four small lamellae + one bristle. Segment 5 of protarsus (Figs 2C, 12F) with two rows of 1 (proximal) + 20 lamellae; distal apex with approximately 14 lamellae. Tibial spurs 1/0/1.

***Male*.** Unknown.

####### Differential diagnosis.

Because of the above diagnosis, the new species is similar to *Gonatopus trochantericus* (Benoit, 1954) and *Gonatopus tulearensis* Olmi, 2010. The main difference regards the metanotum (with sides protruding (rounded protrusions) in *Gonatopus kanyawarus* (Fig. 3A); with sides rounded and not protruding in the other two species (Figs 3D, E)).

####### Etymology.

The species is named after the type locality, Kanyawara.

####### Hosts.

Unknown.

####### Distribution.

Uganda.

###### Gonatopus kolyadai

Taxon classificationAnimaliaHymenopteraDryinidae

Olmi**

Gonatopus kolyadai Olmi, 2007b: 224.

####### Material examined.

***New record*. CENTRAL AFRICAN REPUBLIC: *Sangha-Mbaéré Prefecture***: Reserve Speciale de forêt dense de Dzanga-Sangha, 12.7 km 326°NW Bayanga, 3°00.27'N, 16°11.55'E, 420 m, 11–12.V.2001, Malaise trap, lowland rainforest, S. van Noort leg., 1♀ (SAMC).

####### Hosts.

Unknown.

####### Distribution.

South Africa (Olmi 2007b, 2009). Newly recorded from Central African Republic here.

###### Gonatopus operosus

Taxon classificationAnimaliaHymenopteraDryinidae

Olmi

Gonatopus opacus Olmi, 1984: 1634 (preoccupied).
Gonatopus operosus Olmi, 1993: 80 (new name).

####### Material examined.

***Published record*.** Olmi (1984): **UGANDA**: CENTRAL REGION, Mukono district: Kyagwe (= Kyagur: mistake in original description) [00°25'00"N, 032°50'00"E], XI.1938, T.H.C. Taylor leg., ♀ holotype (BMNH). ***New records*. CAMEROON: *SOUTH-WEST REGION***: 6 mi. S Kumba, 180 m, 1♀ (AMNH). **SOUTH AFRICA: *Western Cape***: Die Dam, 24.II.2006, M. Olmi reared ex *Caffrolix cyclopia* (Cogan), 1♀ (MOLC). **UGANDA: *WESTERN REGION***: Masindi District, Budongo Forest near Sonso, 01°45'N, 31°35'E, VI.1995, Th. Wagner leg., 1♀ (CNCI).

####### Hosts.

Cicadellidae (Guglielmino et al. 2013): in South Africa: *Caffrolix cyclopia* (Cogan).

####### Distribution.

Recorded from Uganda (Guglielmino and Olmi 2007; Olmi 1984). Newly recorded from Cameroon and South Africa here.

###### Gonatopus taylori

Taxon classificationAnimaliaHymenopteraDryinidae

Olmi

Gonatopus taylori Olmi, 1984: 1628.

####### Material examined.

***Published records*.** Olmi (1984): **UGANDA: *EASTERN REGION***: Sironko District, Bugusege (= Bugusaga, misspelt in Olmi 1984) [01°07'20"N, 034°15'55"E], XI.1938, T.H.C. Taylor leg., ♀ holotype (BMNH). ***CENTRAL REGION***: Mubende District, Namutamba, 9.VII.1940, T.H.C. Taylor leg., 1♀ paratype (BMNH); ***EASTERN REGION***: Mayuge district, Bugota [00°20'00"N, 033°37'00"E], 1♀ paratype (AMNH).

####### Hosts.

Unknown.

####### Distribution.

Recorded from Botswana, Ethiopia, Kenya, South Africa and Uganda (Olmi 1984; Olmi and Copeland 2011).

##### Genus *Neodryinus* Perkins

*Neodryinus* Perkins, 1905: 50.

###### Neodryinus antiquus

Taxon classificationAnimaliaHymenopteraDryinidae

Benoit**

Neodryinus antiquus Benoit, 1954: 402.

####### Material examined.

***New records*. CENTRAL AFRICAN REPUBLIC: *Sangha-Mbaéré Prefecture***: Dzanga-Ndoki National Park, Mabéa Bai, 21.4 km 53°NE Bayanga, 3°02.01'N, 16°24.57'E, 510 m, 6.V.2001, sweep, lowland rainforest, marsh clearing, CAR01-S70, S. van Noort leg., 1♀ (SAMC). **DEMOCRATIC REPUBLIC OF THE CONGO: *KATANGA***: Lubumbashi (= Elisabethville), 30.III.1939, 1♀ (IRSN). **ZAMBIA: *LUSAKA PROVINCE***: Lusaka, 17.I.1980, Malaise trap, R.A. Beaver leg., 1♀ (AMNH).

####### Hosts.

Unknown.

####### Distribution.

Madagascar (Benoit 1954; Olmi 1984). Newly recorded from Central African Republic, Democratic Republic of the Congo and Zambia here.

###### Neodryinus tussaci

Taxon classificationAnimaliaHymenopteraDryinidae

Olmi**

Neodryinus tussaci Olmi, 2004b: 179.

####### Material examined.

***New records*. CENTRAL AFRICAN REPUBLIC: *Sangha-Mbaéré Prefecture***: Dzanga-Ndoki National Park, Mabéa Bai, 21.4 km 53°NE Bayanga, 3°02.01'N, 16°24.57'E, 510 m, 4.V.2001, sweep, lowland rainforest, marsh clearing, CAR01-S24, S. van Noort leg., 1♀ (SAMC). **UGANDA: *WESTERN REGION***: Kibale National Park, Kanyawara, Makerere University Biological Field Station, 0°34.390'N, 30°21.658'E, 1587 m, 4–26.VIII.2008, YPT, primary mid-altitude rainforest, near stream, S. van Noort leg., 1♀ (SAMC).

####### Hosts.

Unknown.

####### Distribution.

Cameroon (Olmi 2004b). Newly recorded from Central African Republic and Uganda here.

### Family Embolemidae

#### Genus *Ampulicomorpha* Ashmead

*Ampulicomorpha* Ashmead, 1893: 79.

##### Ampulicomorpha magna

Taxon classificationAnimaliaHymenopteraEmbolemidae

Olmi**

Ampulicomorpha magna Olmi, 1996: 102.

###### Material examined.

***New records*. UGANDA: *WESTERN REGION***: Kibale National Park, Kanyawara, Makerere University Biological Field Station, 0°33.996'N, 30°21.262'E, 1495 m, 3–5.VIII.2005, Malaise trap, secondary mid-altitude rainforest, UG05-M12, S. van Noort leg., 1♂ (SAMC).

###### Hosts.

Unknown.

###### Distribution.

Recorded from Gabon, Kenya, Malawi, South Africa, Zambia and Zimbabwe (Olmi and Copeland 2011). Newly recorded from Uganda here.

##### Ampulicomorpha madecassa

Taxon classificationAnimaliaHymenopteraEmbolemidae

Olmi**

Ampulicomorpha madecassa Olmi, 1999a: 2.

###### Material examined.

***New records*. CENTRAL AFRICAN REPUBLIC: *Sangha-Mbaéré Prefecture***: Dzanga-Ndoki National Park, 38.6 km 173°S Lidjombo, 2°21'60"N 16°03'20"E, 350 m, 21–22.V.2001, Malaise trap, lowland rainforest, CAR01-M172, S. van Noort leg., 1♀ (SAMC).

###### Hosts.

Unknown.

###### Distribution.

Recorded from Madagascar (Olmi 1999a), Kenya and South Africa (Olmi et al. 2015). Newly recorded from Central African Republic here.

#### Genus *Embolemus* Westwood

*Embolemus* Westwood, 1833: 444.

##### Embolemus capensis

Taxon classificationAnimaliaHymenopteraEmbolemidae

Olmi**

Embolemus capensis Olmi, 1997: 141.

###### Material examined.

***New records*. CENTRAL AFRICAN REPUBLIC: *Sangha-Mbaéré Prefecture***: Dzanga-Ndoki National Park, 38.6 km 173°S Lidjombo, 2°21'60"N 16°03'20"E, 350 m, 24–25.V.2001, Malaise trap, lowland rainforest, CAR01-M200, S. van Noort leg., 1♂ (SAMC); same locality label, 24–25.V.2001, CAR01-M204, 2♂♂ (SAMC); same locality label, 23–24.V.2001, CAR01-M189, 3♂♂ (SAMC); same locality label, 23–24.V.2001, CAR01-M193, 1♂ (SAMC); same locality label, 26–27.V.2001, CAR01-M227, 1♂ (SAMC); same locality label, 26–27.V.2001, CAR01-M226, 3♂♂ (1 in SAMC, 2 in MOLC); same locality label, 25–26.V.2001, CAR01-M211, 1♂ (SAMC); same locality label, 25–26.V.2001, CAR01-M212, 1♂ (SAMC); same locality label, 25–26.V.2001, CAR01-M215, 1♂ (SAMC); same locality label, 25–26.V.2001, CAR01-M214, 1♂ (SAMC).

###### Hosts.

Unknown.

###### Distribution.

Recorded from Burundi, Kenya, Madagascar, São Tomé and Principe, South Africa (Olmi 1997, Olmi and Copeland 2011).

### Family Sclerogibbidae

#### Genus *Caenosclerogibba* Yasumatsu

*Caenosclerogibba* Yasumatsu, 1958: 21.

##### Caenosclerogibba probethyloides

Taxon classificationAnimaliaHymenopteraSclerogibbidae

Olmi

Caenosclerogibba probethyloides Olmi, 2005a: 87.

###### Material examined.

***Published records*.** Olmi (2005a): **UGANDA: *WESTERN REGION***: Mbarara District, 25 mi. S Mbarara, 10.XII.1957. Ex *Scelembia*, E. Ross reared, 1 ♀ paratype (CASC).

###### Hosts.

Embiidina (Olmi 2005a): in Cameroon: *Heterembia* sp., *Holembia* sp.; in Ivory Coast: *Nodosembia* sp., *Parachirembia* sp., *Scelembia* sp., unidentified *Embiidae*, unidentified *Teratembiidae*; in Kenya: *Oligotoma saundersii* (Westwood); in Tanzania: unidentified *Teratembiidae*; in Uganda: *Scelembia* sp.

###### Distribution.

Recorded from many Afrotropical countries (Cameroon, Ivory Coast, Kenya, Liberia, Madagascar, Tanzania, Uganda), in addition to Yemen (Olmi 2005a; Olmi et al. 2015).

#### Genus *Probethylus* Ashmead

*Probethylus* Ashmead, 1902: 270.

##### Probethylus callani

Taxon classificationAnimaliaHymenopteraSclerogibbidae

Richards**

Probethylus callani Richards, 1939b: 211.

###### Material examined.

***New records*. CENTRAL AFRICAN REPUBLIC: *Sangha-Mbaéré Prefecture***: Reserve Speciale de Forêt dense de Dzanga-Sangha, 12.7 km 326°NW Bayanga, 3°00.27'N, 16°11.55'E, 420 m, 15–16.V.2001, Malaise trap, lowland rainforest, S. van Noort leg., 2♂♂ (SAMC); Dzanga-Ndoki National Park, 38.6 km 173°S Lidjombo, 2°21'60"N 16°03'20"E, 350 m, 21–22.V.2001, Malaise trap, lowland rainforest, CAR01-M173, S. van Noort leg., 1♂ (SAMC).

###### Hosts.

Embiidina (Olmi 2005a): in the Afrotropical region: Congo: *Parthenembia* sp., *Plesembia* sp., *Dihybocercus collaris*; in Tanzania: *Rhagadochir vosseleri*.

###### Distribution.

Recorded from many Nearctic, Neotropical and Afrotropical countries (Olmi 2005a). In Africa known from Angola, Democratic Republic of the Congo, Kenya, Nigeria, South Africa and Tanzania (Olmi 2005a; Olmi and Copeland 2011; Olmi et al. 2015).

#### Genus *Sclerogibba* Riggio & De Stefani-Perez

*Sclerogibba* Riggio & De Stefani-Perez, 1888: 19.

##### Sclerogibba algerica

Taxon classificationAnimaliaHymenopteraSclerogibbidae

Benoit**

Sclerogibba algerica Benoit, 1963: 84.

###### Material examined.

***New records*. CENTRAL AFRICAN REPUBLIC: *Sangha-Mbaéré Prefecture***: Dzanga-Ndoki National Park, Mabéa Bai, 21.4 km 53°NE Bayanga, 3°02.01'N, 16°24.57'E, 510 m, 1–7.V.2001, YPT, lowland rainforest, marsh clearing, S. van Noort leg., 1♀ (SAMC). **MALI: *Koulikoro Region***: Katibougou, 2008, Malaise trap, D. Sommaggio leg., 1♂ (MOLC).

###### Hosts.

Embiidina (Olmi 2005a): in Algeria: *Embia lesnei* Ross; in Tunisia: *Embia ramburi* (Rimsky-Korsakow). No hosts are known in the Afrotropical region.

###### Distribution.

Recorded from Algeria and Tunisia (Olmi 2005a). Newly recorded from the Afrotropical region (Central African Republic and Mali).

##### Sclerogibba crassifemorata

Taxon classificationAnimaliaHymenopteraSclerogibbidae

Riggio & De Stefani-Perez

Sclerogibba crassifemorata Riggio & De Stefani-Perez, 1888: 146.

###### Material examined.

***Published record*.** Olmi (2005a): **CENTRAL AFRICAN REPUBLIC: *HaUT-MbOMOU Prefecture***: Zemio, 05°45'N, 25°15'E, 6.III.1948, Neal A. Weber leg., 1♂ (AMNH).

###### Hosts.

Embiidina (Olmi 2005a): in Algeria: *Embia lucasi* Ross. No hosts are known in the Afrotropical region.

###### Distribution.

Recorded from many Palaearctic and Afrotropical countries (Olmi 2005a). In Africa known from Central African Republic, Kenya, Niger and Northern Africa (Olmi 2005a; Olmi and Copeland 2011).

##### Sclerogibba impressa

Taxon classificationAnimaliaHymenopteraSclerogibbidae

Olmi

Sclerogibba impressa Olmi, 2005a: 149.

###### Material examined.

***Published record*.** Olmi (2005a): **UGANDA: *CENTRAL REGION***: Wakiso District, Entebbe, 3700’, 14.V.1972, Malaise trap, H. Falke leg., 1♀ paratype (CNCI).

###### Hosts.

Embiidina (Olmi 2005a). No hosts are known in the Afrotropical region. The unique hosts are known in the Philippines and Taiwan (*Aposthonia* species).

###### Distribution.

Recorded from Afrotropical and Oriental countries (Olmi 2005a). In Africa known only from Kenya and Uganda (Olmi 2005a; Olmi and Copeland 2011; Olmi et al. 2015).

##### Sclerogibba rapax

Taxon classificationAnimaliaHymenopteraSclerogibbidae

Olmi**

Sclerogibba rapax Olmi, 2005a: 160.

###### Material examined.

***New records*. CENTRAL AFRICAN REPUBLIC: *Sangha-Mbaéré Prefecture***: Reserve Speciale de Forêt dense de Dzanga-Sangha, 12.7 km 326°NW Bayanga, 3°00.27'N, 16°11.55'E, 420 m, 15–16.V.2001, Malaise trap, lowland rainforest, S. van Noort leg., 2♂♂ (SAMC).

###### Hosts.

Embiidina (Olmi 2005a): in Angola: *Machadoembia* sp., *Scelembia* sp.; in Cameroon: *Plesembia* sp.; in Democratic Republic of the Congo: *Scelembia* sp., *Plesembia* sp.; in Kenya: *Embia* (= *Dictyoploca) burensis* (Rimsky-Korsakov); in Malawi: *Embia* sp.

###### Distribution.

Recorded from many Afrotropical and Oriental countries (Olmi 2005a). In Africa known from Angola, Cameroon, Democratic Republic of the Congo, Ghana. Kenya and Malawi (Olmi 2005a; Olmi and Copeland 2011; Olmi et al. 2015). Newly recorded from Central African Republic.

##### Sclerogibba talpiformis

Taxon classificationAnimaliaHymenopteraSclerogibbidae

Benoit

Sclerogibba talpiformis Benoit, 1950a: 133.

###### Material examined.

***Published records*.** Olmi et al. 2015: **CENTRAL AFRICAN REPUBLIC: *Sangha-Mbaéré Prefecture***: Dzanga-Ndoki National Park, 38.6 km 173°S Lidjombo, 02°21.60'N, 16°03.20'E, 350 m, 24–25.V.2001, lowland rainforest, Malaise trap, S. van Noort leg., CAR01-M206, 1♂ (UKIC). **UGANDA: *WESTERN REGION***: Kibale National Park, Kanyawara, Makerere University Biological Field Station, 0°34.405'N, 30°21.646'E, 1484 m, 16–26.VIII.2008, Malaise trap, primary mid-altitude rainforest, near stream, S. van Noort leg., 1♂ (SAMC).

###### Hosts.

Embiidina (Olmi 2005a): in Ivory Coast: *Parachirembia* sp.; in South Africa: *Embia* sp.; in Zambia: *Embia* sp.

###### Distribution.

Recorded from many countries of the world, excluding the Australian region (Olmi 2005a). In Africa known from Botswana, Burkina Faso, Cameroon, Central African Republic, Democratic Republic of the Congo, Gabon, Ivory Coast, Kenya, Madagascar, Mali, Mozambique, Namibia, South Africa, Uganda, Zambia, Zimbabwe, Yemen and Northern Africa (Azevedo et al. 2010; Olmi 2005a; Olmi and Copeland 2011; Olmi et al. 2015).

##### Sclerogibba vagabunda

Taxon classificationAnimaliaHymenopteraSclerogibbidae

(Bridwell)

Lithobiocerus vagabundus Bridwell, 1919: 36.
Sclerogibba vagabunda (Bridwell): Richards 1939b: 218.

###### Material examined.

***Published records*.** Olmi 2005a: **UGANDA: *WESTERN REGION***: Mbarara District, 10 mi. SE Mbarara, 1300 m, Matured 19.I.1958, E. Ross reared, 1♂ (CASC).

###### Hosts.

Embiidina (Olmi 2005a): in Kenya: *Chirembia* sp., *Gnathembia* sp., *Navasiella* sp., *Cephalembia* sp.

###### Distribution.

Recorded from many countries of the world, excluding the Neotropical Region (Olmi 2005a). In Africa known from Burundi, Kenya, Madagascar, Somalia, Tanzania, Togo, Uganda and Northern Africa (Olmi 2005a; Olmi and Copeland 2011). Known also in Yemen (Olmi 2005a).

## Discussion

The checklist of the Dryinidae, Embolemidae and Sclerogibbidae of Central African Republic and Uganda presented in this paper includes 23 species of Dryinidae, two species of Embolemidae and three species of Sclerogibbidae known from Central African Republic; 39 species of Dryinidae, one species of Embolemidae and four species of Sclerogibbidae known from Uganda. Seventeen species of Dryinidae, two species of Embolemidae and one species of Sclerogibbidae were newly recorded from Central African Republic; sixteen species of Dryinidae and one species of Embolemidae were reported for the first time in Uganda.

With 1827 species worldwide (Olmi and Xu 2015), Dryinidae is one of the three largest families in the Chrysidoidea, the other two being Chrysididae and Bethylidae. With the possible exception of Kenya, Madagascar, Mozambique and South Africa, the dryinid fauna of the Afrotropical Region, like that of many families of micro-Hymenoptera, is poorly known. A comparison with the better known Afrotropical countries shows the following situation: in Madagascar 123 species of Dryinidae, six species of Embolemidae and seven species of Sclerogibbidae are recorded (Azevedo et al. 2010); in South Africa 174 species of Dryinidae (unpublished datum; 119 reported by Olmi (2006)), seven species of Embolemidae (unpublished datum; six reported by Olmi (2006)) and six species of Sclerogibbidae are recorded (Olmi 2005a); in Mozambique 45 species of Dryinidae (Olmi et al. 2012), no species of Embolemidae (unpublished datum) and three species of Sclerogibbidae are known (Olmi et al. 2015); in Kenya 76 species of Dryinidae, six species of Embolemidae and 12 species of Sclerogibbidae are reported by Olmi et al. (2015).

Hosts of Dryinidae are known for 12 of the 52 species reported in the presented checklists in this paper, but no new host associations were established during the recent inventory surveys conducted in Uganda and Central African Republic. No hosts of Embolemidae are known in the entire Afrotropical region. Host associations are better known for the Sclerogibbidae, mainly due to the rearing out of parasitized hosts carried out by Edward S. Ross: the hosts are known for seven of the eight species reported in the presented checklists, in one case as a result of rearing of adult wasps in Uganda.

Further baseline species inventory and establishment of host associations are required across the majority of the countries in the region to enable assessment of overall species richness and biology of the Afrotropical fauna for these families.

## Supplementary Material

XML Treatment for Aphelopus himyarita

XML Treatment for Aphelopus mediocarinatus

XML Treatment for Aphelopus testaceus

XML Treatment for Aphelopus wittei

XML Treatment for Conganteon vulcanicum

XML Treatment for Anteon cautum

XML Treatment for Anteon dzanganum

XML Treatment for Anteon evertsi

XML Treatment for Anteon fisheri

XML Treatment for Anteon granulatum

XML Treatment for Anteon gutturnium

XML Treatment for Anteon hoyoi

XML Treatment for Anteon inflatrix

XML Treatment for Anteon kawandanum

XML Treatment for Anteon kibalense

XML Treatment for Anteon kivuanum

XML Treatment for Anteon makererense

XML Treatment for Anteon mubfs

XML Treatment for Anteon ngoyense

XML Treatment for Anteon semajanna

XML Treatment for Anteon striatum

XML Treatment for Anteon taylori

XML Treatment for Anteon townesi

XML Treatment for Anteon ugandanum

XML Treatment for Anteon whartoni

XML Treatment for Anteon zairense

XML Treatment for Bocchus bini

XML Treatment for Bocchus kibalensis

XML Treatment for Dryinus aethiopicus

XML Treatment for Dryinus erraticus

XML Treatment for Dryinus kibalus

XML Treatment for Dryinus saussurei

XML Treatment for Dryinus shimbanus

XML Treatment for Dryinus turneri

XML Treatment for Dryinus ugandanus

XML Treatment for Dryinus undulatus

XML Treatment for Pseudodryinus townesi

XML Treatment for Echthrodelphax migratorius

XML Treatment for Echthrodelphax tauricus

XML Treatment for Adryinus bellicosus

XML Treatment for Adryinus oweni

XML Treatment for Gonatopus nearcticus

XML Treatment for Gonatopus camerounensis

XML Treatment for Gonatopus guigliae

XML Treatment for Gonatopus hyalinus

XML Treatment for Gonatopus incognitus

XML Treatment for Gonatopus kanyawarus

XML Treatment for Gonatopus kolyadai

XML Treatment for Gonatopus operosus

XML Treatment for Gonatopus taylori

XML Treatment for Neodryinus antiquus

XML Treatment for Neodryinus tussaci

XML Treatment for Ampulicomorpha magna

XML Treatment for Ampulicomorpha madecassa

XML Treatment for Embolemus capensis

XML Treatment for Caenosclerogibba probethyloides

XML Treatment for Probethylus callani

XML Treatment for Sclerogibba algerica

XML Treatment for Sclerogibba crassifemorata

XML Treatment for Sclerogibba impressa

XML Treatment for Sclerogibba rapax

XML Treatment for Sclerogibba talpiformis

XML Treatment for Sclerogibba vagabunda
